# Adolescent self-administration of the synthetic cannabinoid receptor agonist JWH-018 induces neurobiological and behavioral alterations in adult male mice

**DOI:** 10.1007/s00213-022-06191-9

**Published:** 2022-08-09

**Authors:** Giulia Margiani, Maria Paola Castelli, Nicholas Pintori, Roberto Frau, Maria Grazia Ennas, Antonio C. Pagano Zottola, Valeria Orrù, Valentina Serra, Edoardo Fiorillo, Paola Fadda, Giovanni Marsicano, Maria Antonietta De Luca

**Affiliations:** 1 Department of Biomedical Sciences, University of Cagliari, Cagliari, Italy; 2 “Guy Everett” Laboratory, Department of Biomedical Sciences, University of Cagliari, Cagliari, Italy; 3 Institute for Genetic and Biomedical Research, National Research Council (CNR), Lanusei, Italy; 4 INSERM, U1215 NeuroCentre Magendie, Bordeaux, France; 5 University of Bordeaux, Bordeaux, France; 6 Institute of Neuroscience-Cagliari, National Research Council (CNR), Cagliari, Italy; 7 Institut de Biochimie et Génétique Cellulaires, UMR 5095, Bordeaux, France

**Keywords:** Synthetic cannabinoid receptor agonist, Neuroinflammation, GFAP, IBA-1, Cytokines, Chemokines

## Abstract

**Rationale:**

The use of synthetic cannabinoid receptor agonists (SCRAs) is growing among adolescents, posing major medical and psychiatric risks. JWH-018 represents the reference compound of SCRA-containing products.

**Objectives:**

This study was performed to evaluate the enduring consequences of adolescent voluntary consumption of JWH-018.

**Methods:**

The reinforcing properties of JWH-018 were characterized in male CD1 adolescent mice by intravenous self-administration (IVSA). Afterwards, behavioral, neurochemical, and molecular evaluations were performed at adulthood.

**Results:**

Adolescent mice acquired operant behavior (lever pressing, Fixed Ratio 1–3; 7.5 µg/kg/inf); this behavior was specifically directed at obtaining JWH-018 since it increased under Progressive Ratio schedule of reinforcement, and was absent in vehicle mice. JWH-018 IVSA was reduced by pretreatment of the CB1-antagonist/inverse agonist AM251. Adolescent exposure to JWH-018 by IVSA increased, at adulthood, both nestlet shredding and marble burying phenotypes, suggesting long-lasting repetitive/compulsive-like behavioral effects. JWH-018 did not affect risk proclivity in the wire-beam bridge task. In adult brains, there was an increase of ionized calcium binding adaptor molecule 1 (IBA-1) positive cells in the caudate-putamen (CPu) and nucleus accumbens (NAc), along with a decrease of glial fibrillary acidic protein (GFAP) immunoreactivity in the CPu. These glial alterations in adult brains were coupled with an increase of the chemokine RANTES and a decrease of the cytokines IL2 and IL13 in the cortex, and an increase of the chemokine MPC1 in the striatum.

**Conclusions:**

This study suggests for the first time that male mice self-administer the prototypical SCRA JWH-018 during adolescence. The adolescent voluntary consumption of JWH-018 leads to long-lasting behavioral and neurochemical aberrations along with glia-mediated inflammatory responses in adult brains.

**Supplementary Information:**

The online version contains supplementary material available at 10.1007/s00213-022-06191-9.

## Introduction

Adolescence is a critical period in brain development and maturation (Schneider 2013; Manitt et al. 2010; Naneix et al. 2012); this phase of life is an at-risk period for substance use and exposure to drugs, including cannabinoids (Hurd et al. 2014). Therefore, drug abuse propensity during adolescence is higher than in adulthood due to many factors, such as novelty-seeking behavior (Chambers et al. 2003), and a higher sensitivity to the rewarding effects of drugs of abuse (Spear 2016).

The endocannabinoid system, including arachidonoyl ethanol amide (anandamide/AEA), 2-arachidonoylglycerol (2-AG), and cannabinoid receptor 1 (CB1R), achieves maximum expression during adolescence with a subsequent decline at adulthood (Glass et al. 1997; Meyer et al. 2018); this system plays an important role in cognitive and rewarding functions (Koob and Volkow 2010; Parsons and Hurd 2015). Indeed, the EC system modulates dopamine (DA) response to natural reward, as it finely controls the activity of DAergic projections from the ventral tegmental area (VTA) to the nucleus accumbens (NAc), modulating rewarding and reinforcing behaviors (De Luca et al. 2014) and, ultimately, affecting drug use and abuse (Parsons and Hurd 2015).

Synthetic cannabinoid receptor agonists (SCRAs) are the most used novel psychoactive substances (NPS) (Pintori et al. 2017; Miliano et al. 2018; EMCDDA 2019; Musa et al. 2020), particularly among adolescents (Ninnemann et al. 2017), and are posing significant medical and psychiatric risks worldwide (Palamar et al. 2017). Compared to Δ9-tetrahydrocannabinol (THC), the principal psychoactive constituent of Cannabis, SCRAs are extremely potent full agonists of brain CBRs (De Luca et al. 2016), inducing longer-lasting and more adverse effects than Cannabis (Atwood et al. 2010; Cohen and Weinstein 2018). The SCRA JWH-018 (1-pentyl-3-(1-naphthoyl) indole) shares with other drugs of abuse (i.e., ethanol, heroin, cocaine) the ability to stimulate DA release in the NAc shell compared to other DAergic terminal areas arising from VTA (e.g., PFC, NAc core) (Di Chiara 2002, 2004). Recently, in adult rats, we showed that repeated JWH-018 exposure elicits anxious and aversive behaviors, transitory attentional deficits, withdrawal signs, and modifies basal and stimulated DAergic transmission (Pintori et al. 2021). Consistently, SCRA users frequently report adverse effects such as repetitive and compulsive-like behaviors, altered emotional states (Angoa-Pérez et al. 2013), and a propensity for risk-taking behaviors (Clayton et al. 2017).

Glial cells are actively involved in many aspects of central nervous system physiological functions (Araque et al. 2014; Salter and Beggs 2014). They are affected by exposure to different classes of drugs of abuse (Lacagnina et al. 2017; Linker et al. 2018) displaying proliferation, with functional and morphological changes noted in astrocytes and microglia (Ransohoff and Brown 2012; Pocock and Kettenmann 2007). These alterations might contribute to the behavioral outcomes associated with substance abuse (Coller and Hutchinson 2012; Miguel-Hidalgo 2009). Importantly, repeated exposure to JWH-018 during adulthood induces astrogliosis and microgliosis in specific DAergic areas (Pintori et al. 2021).

Similarly to other drugs of abuse, exposure to THC and SCRAs induces alteration in brain cytokine production, leading to either anti-inflammatory (Bhatt et al. 2021; Fields et al. 2020; Tanaka et al. 2020) or pro-inflammatory (Bayazit et al. 2017; Zamberletti et al. 2015) effects. Despite the dramatically increased use of SCRA among young adults (Wagner et al. 2014; Miliano et al. 2018; EMCDDA 2019), their impact on brain function and behavior at adulthood represents an important unanswered health question. In addition, the absence of a reliable adolescent animal model mimicking the voluntarily daily intake of SCRA impedes advancements in the field. Therefore, in this study, we developed an animal model to investigate the reinforcing properties of JWH-018 in male adolescent mice and to evaluate the role of CB1Rs in modulating JWH-018 IVSA. Since growing investigations indicate that SCRA produces compulsive, repetitive, and impulsive behaviors (Mc Donald et al. 2003; Clayton et al. 2017; Mensen et al. 2019), we also investigated these behavioral outcomes in adult mice that self-administered JWH-018 during adolescence by evaluating marble burying, nestlet shredding, and wire-beam bridge tests. In parallel, potential changes in glial cells (astrocytes and microglia) and of cytokines/chemokines (e.g., IL2, IL13, RANTES) expression were investigated at adulthood after adolescent exposure to JWH-018. Lastly, since JWH-018’s activity is CB1R-mediated (De Luca et al. 2015), and passive administration of JWH-018 down-regulates the expression of CB1 receptors in DAergic terminal areas of adult rats (Pintori et al. 2021), we evaluated whether JWH-018 IVSA during adolescence can induce long-term alterations in the expression of CB1Rs in brain areas involved in addiction and motivated behavior, such as the cortex and striatum.

## Materials and methods

### Animals

Adolescent CD1 male mice (Envigo, Netherlands), weighing 22–27 g, were used from postnatal day (PND) 25 to 80 for intravenous self-administration and subsequent behavioral and neurochemical studies. Mice were housed in groups of three to six per cage with standard conditions of temperature (21 ± 1 °C) and humidity (60%) under a 12-h light/dark cycle (lights on at 7.00 AM) and with ad libitum access to water and food (Mucedola, Italy) until the intravenous self-administration (IVSA) experiments. All animal care procedures and experiments were carried out in accordance with European Council directives (609/86 and 63/2010) and in compliance with the animal policies issued by the Italian Ministry of Health and the Ethical Committee for Animal Experiments (CESA, University of Cagliari). We made all efforts to minimize pain and suffering and to reduce the number of animals used, according to the 3Rs principles.

### Drugs

JWH-018, AM251, and AM630 were purchased from Tocris (Bristol, UK). Drugs were solubilized in 0.5% EtOH, 0.5% Tween 20, and 99% saline. AM251 and AM630 (CBR1- and CBR2-antagonist/inverse agonists, respectively) were administered intraperitoneally (i.p.) 30 min prior to JWH-018 IVSA at different doses depending on the group of animals; AM251: 0.3–1.0 mg/kg (volume 10 mL/kg); AM630: 0.5 and 1.0 mg/kg (volume 10 mL/kg).

### Overall experimental design and study group

**Fig. 1:**
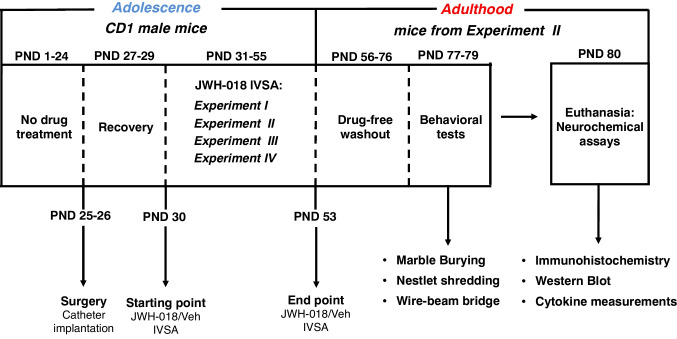
Overall experimental design. Groups of male CD1 mice studied by different experimental procedures, during adolescence and adulthood. IVSA, intravenous self-administration; PND, postnatal day. JWH-018 IVSA: Experiment I (*n* = 8 per group); Experiment II (JWH-018 *n* = 10; Veh *n* = 15); Experiment III (*n* = 7); Experiment IV (*n* = 5). Behavioral tests: marble burying and nestlet shredding (*n* = 5 per group); wire-beam bridge (*n* = 5 per group). Neurochemical assays: immunohistochemistry (*n* = 5 per group); Western Blot and cytokine measurements (JWH-018 *n* = 5; Veh *n* = 10)

This study was designed to characterize the behavioral and neurobiological consequences of adolescence JWH-018 IVSA at adulthood (Fig. 1). First, we performed IVSA experiments in adolescent mice (PND 30–55) to assess (i) the JWH-018 dose–response curve (Experiment I); (ii) the response rates in different reinforcement IVSA schedules (FR1-FR3-PR; Experiment II); (iii) the response rates in different JWH-018 IVSA phases (Acquisition, Extinction, Reacquisition; Experiment III), and (iv) the effect of CBRs blockade on JWH-018 IVSA (Experiment IV). Afterwards, mice from Experiment II (IVSA from PND 34 to 53) were used for behavioral and neurochemical characterization at adulthood. In particular, after 3 days of recovery from surgery (PND 31–33), IVSA experiment started under different schedules of reinforcement from PND 34 to 53 for 7 days/week. At adulthood, after a drug-free washout period (~ 3 weeks), from PND 77 to 79 animals were randomly divided into different groups to perform behavioral tests, with a sub-group of the mice of each treatment group assigned to repetitive/compulsive-like behaviors’ evaluation (marble burying and nestlet shredding tests) and another sub-group assigned to risk-taking behaviors’ evaluation (wire-beam bridge). At PND 80, mice were sacrificed for subsequent neurochemical assays. In particular, a sub-group of the mice of each group were randomly assigned to immunohistochemical evaluations (GFAP and IBA-1 immunoreactivity), and another sub-group to western immunoblotting (COX-2, EAAT2, CB1 expression) and cytokines measurements. The experimental group sizes (n ≥ 5) were chosen based on our previous experimental protocols (Pintori et al. 2021; Castelli et al. 2014) and are shown in the figure legends. Due to experimental protocol criteria (e.g., acquisition criteria during IVSA phase) and/or technical issues (e.g., catheter obstruction or damage), some animals were excluded from statistical analysis, thus reducing group sizes in few cases.

### Intravenous self-administration studies during adolescence

Detailed descriptions of surgery and IVSA methods are included in the Supplementary Information (SI).

#### Characterization of JWH-018 dose–response curve in the IVSA experimental paradigm (Experiment I)

The first experiment was aimed at characterizing a dose–response curve of JWH-018 (2.5–15 µg/kg/25 µl infusion) in adolescent mice. Mice IVSA experiment was carried out under Fixed Ratio (FR) 1 schedule of reinforcement (1 right lever press: 1 injection; 20-s time-out with light off, session duration 2 h), from PND 30 until PND 53 (24 sessions). During time-out (TO) period, right (active) lever presses had no programmed consequences, while left (inactive) lever presses were never associated with programmed consequences.

#### Characterization of response rates in different reinforcement IVSA schedules (FR1, FR3, PR; Experiment II)

Once the dose at which adolescent mice acquired operant behavior was established (7.5 µg/kg/25 µl infusion), we used different FR protocols to evaluate the reinforcing properties of JWH-018. After a first acquisition phase under FR1 schedule of reinforcement, in which the number of lever pressing achieved 80% of stability for at least three consecutive sessions (7 sessions in total), mice underwent FR3 (3:1) for 12 sessions. The same protocol of IVSA was used for the control group, which was exposed to Vehicle solution (0.5% EtOH, 0.5% Tween 20, and 99% saline). During the last session of the JWH-018 IVSA, after at least 75% responding on the active lever in three consecutive FR3 sessions, the experiment was performed under a Progressive Ratio (PR) schedule of reinforcement in which the number of active lever presses required to obtain each subsequent injection was based on the adapted exponential sequence: 1, 2, 4, 6, 9, 12, 15… (Valentini et al. 2013). PR sessions lasted for 2 h or until mice did not complete the ratio for the delivery of at least one injection within 1 h.

#### Characterization of response rates in different JWH-018 IVSA phases (Acquisition, Extinction, Reacquisition; Experiment III)

After the acquisition phase under FR1-FR3 schedules (see acquisition criteria), a different group of mice underwent extinction and reacquisition phase protocols to assess seeking behavior and reacquisition of operant behavior after the extinction phase to JWH-018. Extinction session schedule was identical to the acquisition schedule, except for the absence of any delivery of JWH-018 infusion. After 6 sessions performed in extinction phase, the protocol was switched to reacquisition phase for 7 sessions in the same condition of acquisition.

#### Effect of CBRs blockade on JWH-018 IVSA (Experiment IV)

To investigate the role of CBRs on operant behavior, in two separate groups of animals subjected to the same protocol of the *Experiment II* (except for PR), we evaluated the effects of the CB1R antagonist/inverse agonist AM251 (0.3–1.0 mg/kg i.p.), and the CB2R antagonist/inverse agonist AM630 (0.5, 1.0 mg/kg i.p.) injections on IVSA behavior. AM251, AM630, or vehicle has been administered 30 min before starting one of the IVSA FR3 session that reached the established criteria of stability (see acquisition criteria); before each antagonist pretreatment, responsiveness for JWH-108 was established to the same stability criteria.

### Behavioral studies at adulthood

For the behavioral studies, after a drug-free period (~ 3 weeks) from the last session of IVSA, adult mice (PND 77–79) from *Experiment II* were tested in marble burying, nestlet shredding and wire-beam bridge tests to assess potential repetitive/compulsive-like and risk-taking phenotypes, respectively (Fig. 1). To minimize stress carry-over effects, mice were subjected to these behavioral tasks in the same experimental room (light intensity between 20 and 30 lx), with an inter-test interval of 24 h and performed in the following order: marble burying, nestlet shredding, and wire-beam bridge tests.

#### Marble burying test

Marble burying test was performed in a transparent plastic cage (50 cm L, 30 cm W, 20 cm H) containing 5 cm of fresh hardwood chip bedding. Twenty standard glass marbles (1.5 cm in diameter, arranged in five rows of four marbles each) were placed uniformly over the surface of bedding. Mice were individually placed in the cage and their activity was recorded for 20 min. At the end of the session, animals were gently removed, and the number of marbles totally (≥ 95%) buried was counted. In order to avoid the presence of olfactory cues, bedding was replaced and marbles were cleaned between each mouse test.

#### Nestlet shredding

Nestlet shredding test was performed in the same type of cage used for marble burying, but with 3 cm of fresh hardwood chip bedding containing a 5 × 5 cm packed cotton nestlet (Ancare Corp; Bellmore, NY) laid on the top of the bedding material. Mice were individually placed in the cage and their activity was monitored for 75 min. Then, the remaining cotton nestlet was dried overnight and the amount (i.e., percentage) of the nestlet shredded was determined by weighing the day after.

#### Wire-beam bridge

This test was performed as previously described, with slight modifications (Frau et al. 2017). The apparatus consisted of 50-cm high Plexiglas platform and a 100-cm high Plexiglas wall, oppositely placed at 50 cm distance. Platforms were connected by a horizontal, flexible wire-mesh metallic grid. Mice were individually placed in the proximity of the edge (3 cm from the edge) of one platform, to make the starting position uncomfortable and promote movement. Behavioral measures, as index of behavioral disinhibition, were scored for 5 min and included the latency to access the bridge (with all 4 paws on it) and to first movement; movement duration and frequency; stretch attend postures and head dipping phenotypes.

### Immunohistochemistry

At the end of the behavioral tests (PND 80), a sub-group of the animals from the *Experiment II* were selected for immunostaining analyses, while another sub-group for cytokines measurements and western immunoblotting (see Fig. 1).

#### Brain tissue preparation and GFAP and IBA-1 immunofluorescent staining

Procedures were carried out as previously described (Castelli et al. 2014; Pintori et al. 2021) (for details, see SI).

#### Imaging and quantitative analysis of GFAP and IBA-1 immunofluorescent staining

An Olympus IX 61 microscope and an Olympus 12-bit cooled F View II camera (Hamburg, Germany) were used for observations and for capturing the images, respectively (For details, see SI).

### Cytokine measurements and Western immunoblotting

#### Sample processing

Mice were sacrificed by cervical dislocation; striatum and cortex were quickly collected and snap frozen in liquid nitrogen and stored at − 80 °C. For cytokine measurements and immunoblotting for excitatory amino acid transporter [(EAAT2) or glutamate transporter (GLT-1)] and cyclooxygenase 2 (COX-2), tissues were prepared following the instruction of Bio-Plex Cell Lysis Kit Product Insert (for details, see SI).

#### Cytokine measurements

Brain cytokine concentrations were measured using a fully quantitative ELISA-based chemiluminescent assay (BioRad 23-plex mouse kit), which simultaneously detects cytokines, chemokines, and growth factors. All samples were run in duplicate and assayed with the BioRad reagent kit and cell lysis for tissue samples, according to the manufacturer’s instructions. Lyophilized cytokines standards containing cytokines: [(IL)1α, IL1β, IL2, IL3, IL4, IL5, IL6, IL9, IL10, IL12(p40), IL12(p70), IL13, IL17, interferon (IFN)γ, TNFα; chemokines: eotaxin, monocyte chemotactic protein-1 (MCP-1), macrophage inflammatory protein (MIP)1α, MIP1β, regulated on activation normal T cells expressed and secreted (RANTES), keratinocyte derived chemokine (KC/GRO/CXCL1), and growth factors: granulocytes macrophage colony-stimulating factor (GM-CSF) and granulocyte (G)-CSF)] were reconstituted to a master standard stock (for details, see SI).

#### EAAT2, COX-2, and CB1R immunoblotting

Samples (cortex and striatum) were thawed on ice and diluted to final concentration of 12 µg/µl protein.

Samples with equal amounts of protein (60 µg) mixed with Laemmli loading buffer were denatured at 70 °C for 10 min and loaded on gradient gel (4–12% NuPAGE Bis–Tris mini gels 15 well 1 mm gels, Life Technologies, CA, USA). To identify the specific bands, internal molecular weight (MW) standards (Precision Plus Protein Western C Standards, Bio-Rad, Hercules, CA, USA) were run in parallel. Then, proteins were transferred onto polyvinylidene difluoride (PVDF) membranes following the company’s protocol (Amersham GE Healthcare, UK). The protein transfer was checked by the ponceau red coloring of the membrane. Membranes were blocked for 1 h at room temperature using a mixture of 20 mM Tris base, 137 mM sodium chloride, and 0.1% Tween 20 (TBS-T) containing 5% BSA or dry powder milk before incubation overnight at 4 °C with the primary antibodies. The following primary antibodies were used: mouse monoclonal anti-EAAT2 (1:500; SC-365634; Santa Cruz, Dallas, TX), mouse monoclonal anti-COX-2 (1:100; SC-376861; Santa Cruz, Dallas, Texas, USA), and mouse monoclonal anti-β-Actin (1:2000; mAbcam 8226; Abcam, Cambridge, UK). For detection of COX-2, the blot was stripped with Restore Western Blot Stripping Buffer (Thermo Scientific, Rockford, IL) and re-blotted with the anti-COX-2 overnight at 4 °C. Blots were incubated with goat horseradish peroxidase (HRP) conjugated secondary anti-mouse antibody (1:5000; Vector, CA, USA) for 1 h at RT, and after TBS-T wash, incubated with the chemiluminescent detection solution Clarity Western ECL Substrate (Bio-Rad, Hercules, CA, USA) according to the protocol provided by the company, and visualized by ImageQuant LAS-4000 (GE Healthcare, Little Chalfont, UK). The signals of the specific bands were normalized with the densities of the corresponding band of endogenous protein β-Actin. Therefore, the normalized data were expressed as a protein/β-actin ratio. Densitometric analysis of the acquired signals was carried out, using the Image Studio Lite Software (LI-COR Biosciences (RRID:SCR_014211, Li-Cor, http://www.licor.com/bio/products/software/image_studio_lite/). With regard to CB1R immunoblotting, protein extracts were mixed with denaturing 4 × Laemmli loading buffer and warmed for 30 min at 37 °C. Samples (18–24 µg per lane) were analyzed on 4–20% precast polyacrylamide gels (Bio-Rad, Hercules, California) and transferred onto PVDF membranes 0.45 µm (Merk Millipore, Billerica, MA). Membranes were blocked in a mixture of Tris-buffered saline and polysorbate 20 (20 mM Tris–HCl pH 7.6, 150 mM NaCl, 0.05% Tween 20) containing 5% of non-fat milk for 1 h at RT. The membrane was incubated at RT for 1 h using antibodies rabbit anti-CB1R (CB1, ab23703; 1:200, Abcam, Cambridge, UK), mouse anti-alpha tubulin (used as loading control) (sc69969; 1:5000, Santa Cruz, Dallas, US). After the transfer step, the membranes were incubated for 1 h with the primary antibody anti-CB1R. After stripping with Restore Western Blot Stripping Buffer, membranes were re-blotted with a mouse monoclonal anti-alpha-tubulin antibody for normalization. Bound primary antibodies were detected with HRP-linked antibodies (1:2000, Cell Signaling Technology, Danvers, MA) and visualized by enhanced chemiluminescence detection (Clarity Western ECL Substrate, Bio-Rad, Hercules, California). The optical densities of immunoreactive bands were quantified by the Image Lab software (Bio-Rad, Hercules, California) after acquisition on ChemiDoc Touch (Bio-Rad, Hercules, California, US).

### Statistical analysis

All data are presented as mean ± SEM. Data were tested for normal distribution using Shapiro–Wilk’s test. Non-parametric test (i.e., Mann–Whitney *U*-test) was chosen when data were found not to be normally distributed. For IVSA studies, the response rates exhibited during each IVSA phase (Acquisition, Extinction, Reacquisition) schedule (FR1-FR3), and JWH-018 dose tested (2.5–15 µg/kg/25 µl infusion) were analyzed separately by two-way repeated measures (RM) ANOVA with response (i.e., active or inactive lever presses), session and/or schedule (mean of the last 3 FR3 sessions, PR) as factors, followed by Sidak’s multiple comparisons. For RM tests, whenever we could not assume sphericity, a Geisser-Greenhouse correction was carried out by GraphPad Prism 8 software (GraphPad Prism). Considering JWH-018 intake, the data (mean ± SEM of JWH-018 consumption at each dose during 2 h of IVSA sessions) were analyzed by RM one-way ANOVA followed by Sidak’s post hoc test. The same statistical analysis was used to assess the effects of CBRs blockade on IVSA. For neurochemical assays, to assess the effect of JWH-018 IVSA on cytokine levels and on GFAP and IBA-IR, data were analyzed by using two-way ANOVA, with treatment and brain areas as factors, followed by Tukey’s multiple comparison. Then, the effect of IVSA on cytokines, GFAP, and IBA-1-IR within each brain area was analyzed by Student’s *t*-test. For behavioral and Western Blot experiments, the data were analyzed by using Student’s *t*-test. Differences were considered significant at *p* < 0.05. Effect sizes were calculated by using Cohen’*d* or Hedges’*g* when sample sizes were equal or not equal, respectively*. *Post hoc* tests were conducted* only when a significant main effect and/or interaction were detected. All analyses were performed using the GraphPad software package (Prism, version 8; GraphPad, San Diego, California, USA).

## Results

### Adolescent IVSA studies

#### Characterization of JWH-018 dose–response curve in the IVSA experimental paradigm (Experiment I)

**Fig. 2:**
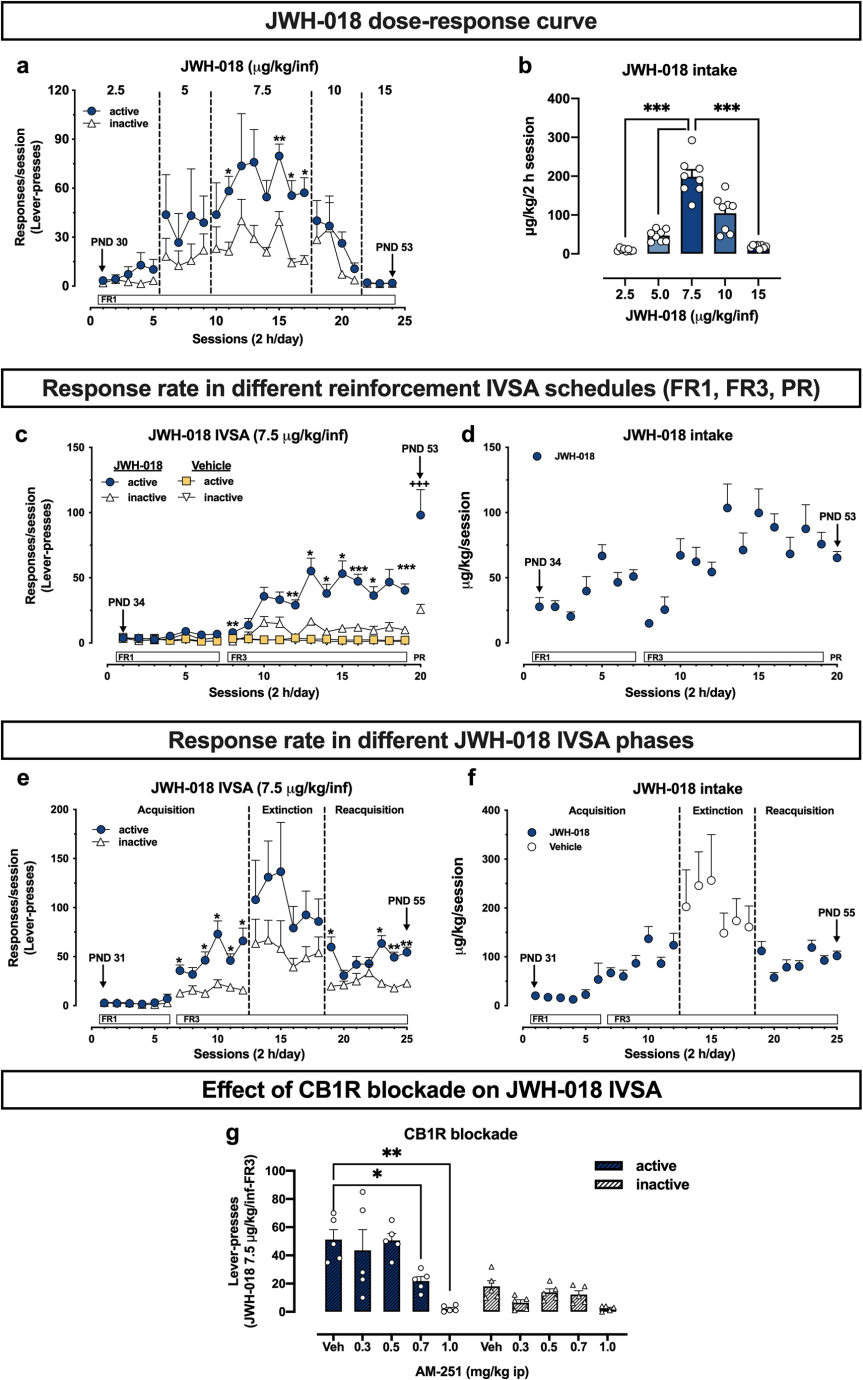
Characterization of JWH-018 IVSA in adolescent mice. (**a**) JWH-018 dose–response curve in the IVSA experimental paradigm (*Experiment I*). Results are expressed as mean ± SEM of the number of active (*circles*) and inactive (*triangle*) lever presses exhibited during each 2 h daily session under FR1 reinforcement schedules at different JWH-018 doses (2.5–15 µg/kg/inf). *p < 0.05, ** *p* < 0.01 vs inactive lever presses (two-way ANOVA; Sidak’s post hoc test); *N* = 8 per group. (**b**) Daily intake during JWH-018 IVSA dose–response curve. Data are expressed as µg/kg of JWH-018 self-administered during each 2 h daily session. Each bar represents the mean ± SEM of the drug self-administered at each dose during the IVSA sessions as indicated in the panel A, *p < 0.05, **** *p* < 0.0001 vs all the other groups (one-way ANOVA; Sidak’s post hoc test); *n* = 8 per group. (**c**, **d**) Response rates in different reinforcement IVSA schedules (FR1-FR3-PR; *Experiment II*). Results are expressed as mean ± SEM of the number of active (*circles*) and inactive (*triangle*) lever presses exhibited during each 2 h daily session under FR1, FR3, or PR reinforcement schedules during JWH-018 (7.5 µg/kg/inf) or vehicle (25 µl/inf) IVSA. *p < 0.05, ** *p* < 0.01, *** *p* < 0.001 vs inactive lever presses; ^+++^ *p* < 0.001 vs FR3 session (mean of the last 3 sessions) (two-way ANOVA; Sidak’s post hoc test); JWH-018: *N* = 10; Veh: *n* = 15. (**e**) Response rates in different JWH-018 IVSA phases (Acquisition, Extinction, Reacquisition; *Experiment III*). Results are expressed as mean ± SEM of the number of active (*circles*) and inactive (*triangle*) lever presses exhibited during each 2 h daily session during acquisition, extinction, and reacquisition phases of JWH-018 IVSA (7.5 µg/kg/inf). *p < 0.05, ** *p* < 0.01 vs inactive lever presses (two-way ANOVA; Sidak’s post hoc test); *n* = 7 per group. (**f**) Effect of CB1R blockade on JWH-018 IVSA (*Experiment IV*). Results are expressed as mean ± SEM of the number of active (*black bars*) and inactive (*white bars*) lever presses exhibited 30 min after intraperitoneal injection of vehicle or AM251 (0.3–1.0 mg/kg). *p < 0.05 ** *p* < 0.01 vs Vehicle (Veh) (one-way ANOVA; Sidak’s post hoc test); *n* = 5 per group

To characterize the pattern of response of CD1 male adolescent mice (PND 30–53), we investigated the JWH-018 dose–response curve using the IVSA experimental paradigm. Based on previous studies performed in adult C57 mice (De Luca et al. 2015), we chose a range of doses of JWH-018 from 2.5 to15 µg/kg/25 µl infusion (lever pressing, FR 1:1). Two-way ANOVA analysis (response × session) performed for the dose of 2.5, 5, 10, and 15, revealed no significant main effects or interaction. Only at the dose of 7.5 µg/kg/inf, two-way ANOVA showed a main effect of response (*F*_(1,14)_ = 11.37, *p* = 0.0046). Sidak’s post hoc test of the response main effect showed a significant difference between active and inactive lever presses in the 11^th^ (*p* < 0.05, Cohen’ *d* = 1.7421), 15^th^ (*p* < 0.01, Cohen’ *d* = 2.1085), 16^th^ (*p* < 0.05, Cohen’ *d* = 2.1583), and 17^th^ sessions (*p* < 0.05, Cohen’ *d* = 2.1456) (Fig. 2(a)). Consistently, RM one-way ANOVA analysis revealed a significant effect of JWH-018 dose (*F*_(1.66,11.65)_ = 48.68, *p* < 0.0001). Sidak’s post h*oc* test showed higher JWH-018 intake at the dose of 7.5 µg/kg/inf as compared to the doses of 2.5 (*p* < 0.0001, Cohen’ *d* = 5.3309), 5.0 (*p* < 0.001, Cohen’ *d* = 4.1178), and 15 (*p* < 0.001, Cohen’ *d* = 5.0814) µg/kg/inf (Fig. 2(b)).

#### Characterization of response rates in different reinforcement IVSA schedules (FR1-FR3-PR; Experiment II)

Once the dose at which adolescent mice acquired operant behavior was established (7.5 µg/kg/25 µl infusion), we used different schedules of reinforcement (Fixed and Progressive Ratio) to evaluate the reinforcing properties of JWH-018. Under FR1 schedule, two-way ANOVA analysis (response × session) revealed no significant main effects or interaction. On the contrary, under FR3 schedule, two-way ANOVA analysis showed a main effect of response (*F*_(1,18)_ = 51.88, *p* < 0.0001), of session (*F*_(3.42,61.64)_ = 7.02, *p* = 0.0002), and of response × session interaction (*F*_(11,198)_ = 2.70, *p* = 0.0029). Sidak’s post hoc test revealed significant differences between active and inactive lever presses in the 8^th^ (*p* < 0.01, Cohen’ *d* = 2.4539), 12^th^ (*p* < 0.01, Cohen’ *d* = 2.5103), 13^th^ (*p* < 0.05, Cohen’ *d* = 1.6962), 14^th^ (*p* < 0.05, Cohen’ *d* = 1.7843), 15^th^ (*p* < 0.05, Cohen’ *d* = 1.8585), 16^th^ (*p* < 0.001, Cohen’ *d* = 2.7341) 17^th^ (*p* < 0.05, Cohen’ *d* = 1.5941), and 19^th^ (*p* < 0.001, Cohen’ *d* = 2.5379) sessions (Fig. 2(c)). Considering differences between FR3 and PR schedule, two-way ANOVA analysis showed a main effect of response (*F*_(1,18)_ = 20.45, *p* = 0.0003), of schedule (*F*_(1,18)_ = 15.43, *p* = 0.0010), and of response × schedule interaction (*F*_(1,18)_ = 5.23, *p* = 0.034). Sidak’s post hoc test revealed a significant increase of active but not inactive lever presses during PR session as compared to FR3 session (*p* < 0.001, Cohen’ *d* = 1.2647). Consistently, a control group of mice failed to acquired Vehicle IVSA both under FR1 and FR3 schedule, confirming that the operant behavior was specifically directed at obtaining JWH-018 (Fig. 2(d)).

#### Characterization of response rates in different JWH-018 IVSA phases (Acquisition, Extinction, Reacquisition; Experiment III)

To further assess the abuse potential of JWH-018, we evaluated seeking behaviors for JWH-018 through the extinction phase and the following reacquisition of operant behavior. Under FR1 schedule, two-way ANOVA analysis (response × session) revealed no significant main effects or interaction. On the contrary, under FR3 schedule, two-way ANOVA analysis showed a main effect of response (*F*_(1,12)_ = 15.84, *p* = 0.0018), of session (*F*_(2.83,34.02)_ = 9.19, *p* = 0.0002), and of response × session interaction (*F*_(5,60)_ = 5.18, *p* = 0.0005). Sidak’s post hoc test revealed significant differences between active and inactive lever presses in the 7^th^ (*p* < 0.05, Cohen’ *d* = 2.0024), 9^th^ (*p* < 0.05, Cohen’ *d* = 1.989), 10^th^ (*p* < 0.05, Cohen’ *d* = 1.9389), 11^th^ (*p* < 0.05, Cohen’ *d* = 1.9257), and 12^th^ (*p* < 0.05, Cohen’ *d* = 1.995) sessions. Again, no difference in lever responding were observed during extinction phase, while two-way ANOVA analysis during reacquisition showed a main effect of response (*F*_(1,12)_ = 27.57, *p* = 0.0002) and of response × session interaction (*F*_(6,72)_ = 2.83, *p* = 0.016). Sidak’s post hoc test revealed significant differences between active and inactive lever presses in the 19^th^ (*p* < 0.05, Cohen’ *d* = 2.0057), 23^th^ (*p* < 0.05, Cohen’ *d* = 2.4367), 24^th^ (*p* < 0.01, Cohen’ *d* = 2.7885), and 25^th^ (*p* < 0.01, Cohen’ *d* = 2.8090) sessions (Fig. 2(e)).

#### Effect of CBRs blockade on JWH-018 IVSA (Experiment IV)

To investigate the involvement of CB1R on operant behavior, we studied the effect of the administration of the selective antagonist/inverse agonist AM251 (0.3–1.0 mg/kg i.p.; 30 min before the IVSA FR3 session) on JWH-018 (7.5 µg/kg/25 µl infusion) IVSA behavior. RM one-way ANOVA analysis revealed a significant effect of AM251 dose (*F*_(1.23,4.92)_ = 12.54, *p* = 0.0015). Sidak’s post hoc test showed a significant decrease of active lever presses at the dose of 0.7 (*p* < 0.05, Cohen’ *d* = 2.4027) and 1.0 (*p* < 0.01, Cohen’ *d* = 4.3616) mg/kg of AM251 as compared to vehicle (Fig. 2(f)). On the other hand, data showed that the administration of the CB2R antagonist/inverse agonist AM630 (0.5 and 1.0 mg/kg i.p.) did not affect IVSA operant behavior (Supplementary Fig. 1S).

### Behavioral studies at adulthood

#### Marble burying and nestlet shredding test

**Fig. 3:**
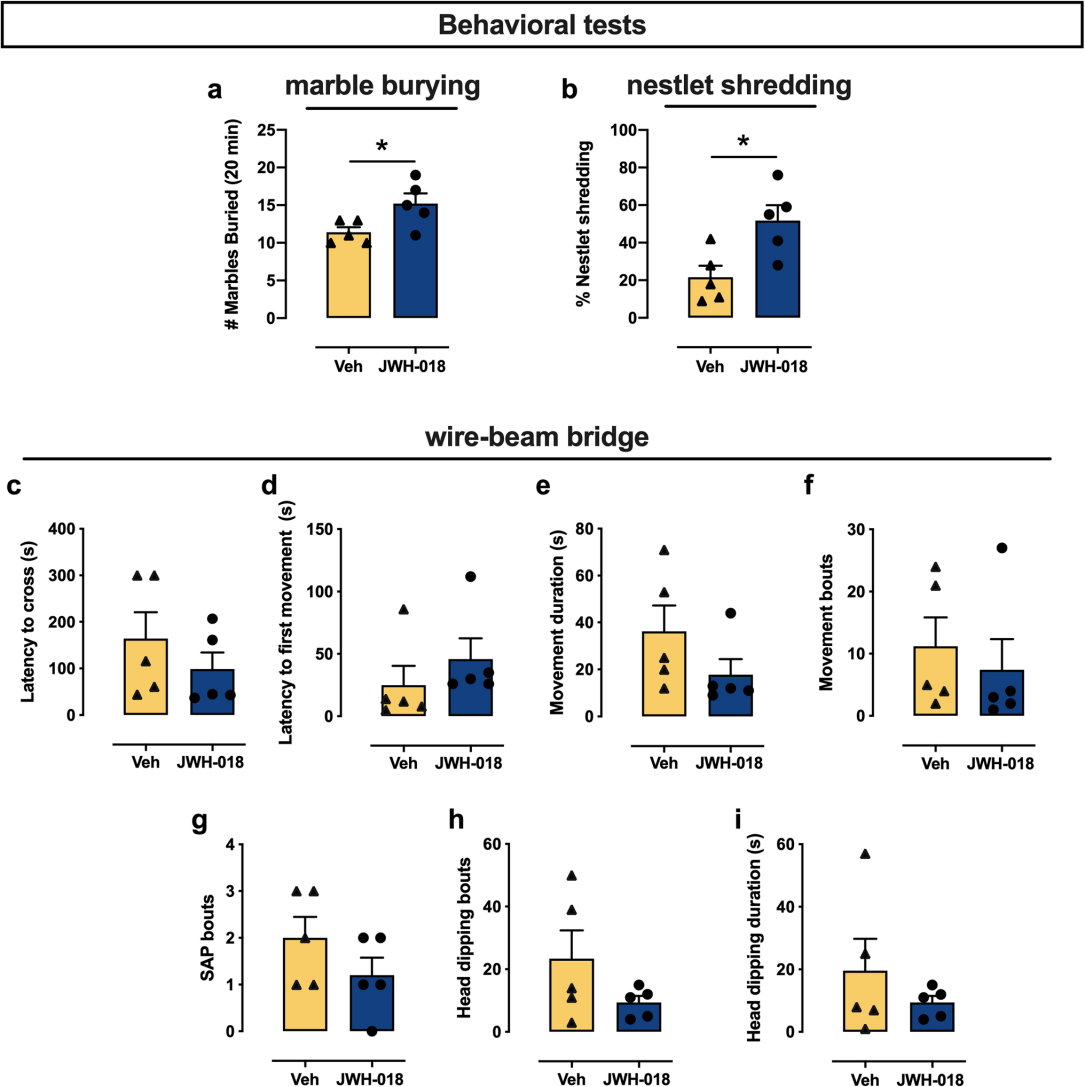
Long-term behavioral effects of JWH-018 IVSA in marble burying (MB) (**a**), nestlet shredding (**b**), and wire-beam bridge (**c**–**i**) tests. Results are expressed as mean ± SEM (**a**) of the number of marbles buried in the MB test, (**b**) of the percentage of nestlets shredded in the nestlet shredding test, (**c**, **d**) of the latency (sec) to cross the bridge and to first movement, (**e**, **f**) of the movement duration (sec) and bouts, (**g**, **h**) of the number of stretch attend postures (SAP) and head dipping bouts, and (**i**) of the head dipping duration (sec) in the wire-beam bridge test. * *p* < 0.05 vs. vehicle-treated mice; (unpaired Student’s *t*-test); *n* = 5 per group

The marble burying test showed that adolescent exposure to JWH-018 increased the number of completely covered marbles compared to vehicle group (*t*_(8)_ = 2.51; *p* = 0.036; + 33%, Cohen’ *d* = 1.5874; Fig. 3(a)). Consistently, nestlet shredding test showed that adolescent exposure to JWH-018 increases nestlet shredding compared to their vehicle counterparts (*t*_(8)_ = 2.97; *p* = 0.018; + 140%, Cohen’ *d* = 1.8767; Fig. 3(b)). These data suggest that JWH-018 self-administration during adolescence exerted permanent, long-lasting repetitive/compulsive-like behavioral effects at adulthood.

#### Wire-beam bridge

In contrast with marble burying and nestlet shredding data, repeated exposure to JWH-018 during adolescence failed to modify a number of risk-taking behaviors in the wire-beam bridge task assessed at adulthood. Indeed, adult JWH-018 mice and their vehicle counterparts exhibited similar risk-assessment phenotypes when positioned on an unstable suspended bridge. Accordingly, analyses showed no significant differences in the latency to cross the bridge as well as to the first movement, duration, and number of movements, number of stretch-attend posture episodes, duration, and number of head dipping (Fig. 3(c)-(i)).

### Immunohistochemistry and neurochemical studies

#### Adolescent JWH-018 IVSA results in long-term changes of IBA-1 and GFAP immunoreactivity in specific brain areas

**Fig. 4:**
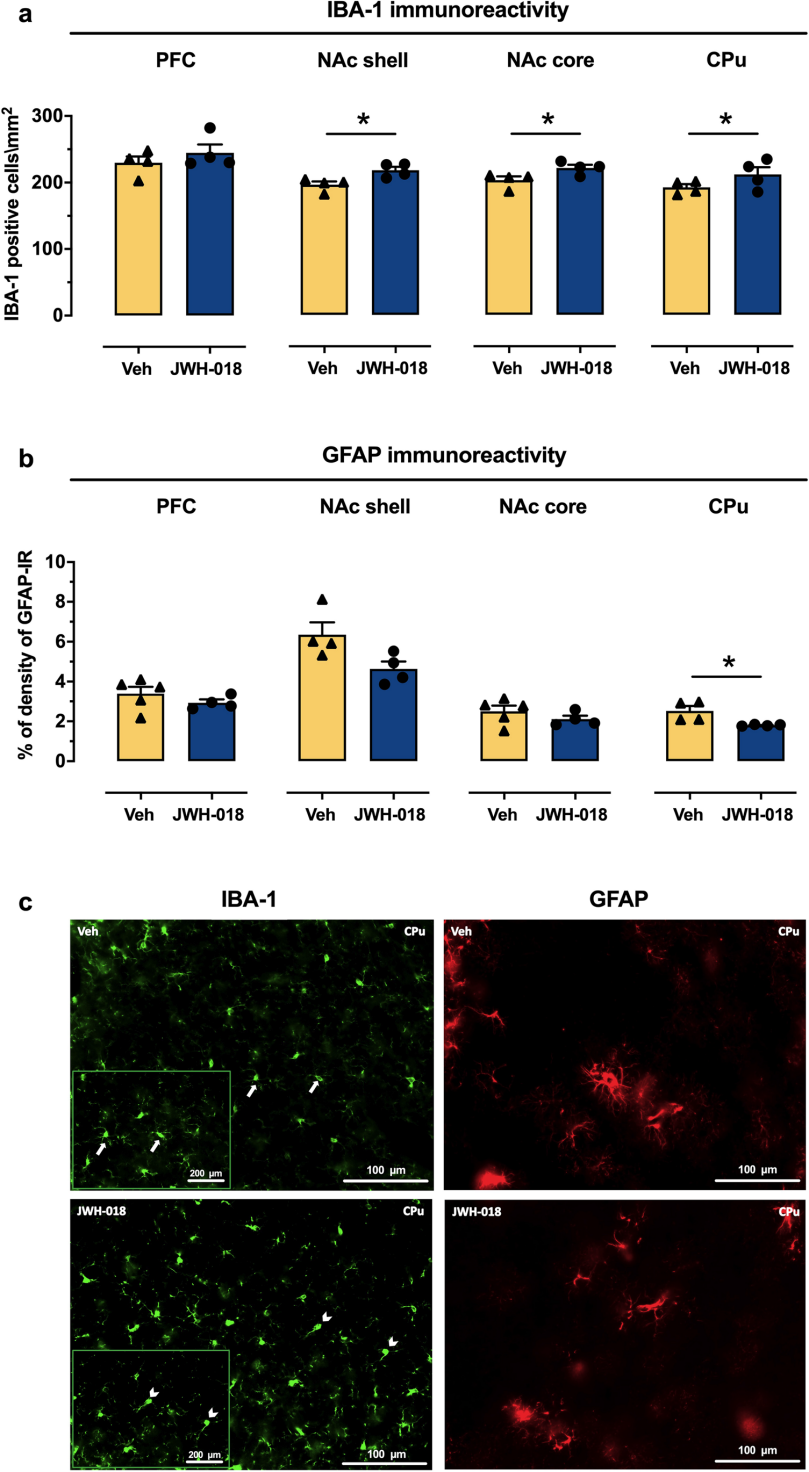
Long-term effects of JWH-018 IVSA on IBA-1 and GFAP immunoreactivity in specific brain areas. Adolescent JWH-108 IVSA at adulthood: (**a**) increased the number of IBA-1 positive cells in the dorsal CPu and in the NAc core and shell; *p < 0.05 compared to Vehicle (Veh) group (unpaired Student’s *t*-test); *N* = 4 per group, and (**b**) decreased GFAP immunoreactivity in the CPu, with a downward trend observed in the other brain areas; JWH-018: *n* = 4 per group; Veh: *n* = 5 (PFC, NAc core), *n* = 4 (CPu, NAc shell). Values represent mean ± SEM of number of IBA-1 positive cells/mm^2^ or percentage of GFAP-IR density; *p < 0.05 compared to Vehicle (Veh) group (unpaired Student’s *t*-test). (**c**) Representative images (20 × magnification) of IBA-1 and GFAP immunostaining in the CPu of adult mice that underwent JWH-018 or Veh IVSA during adolescence. Note the resting microglia (arrows, resting state in the upper left panel) and the amoeboid microglia (arrowhead, activated state in the lower left panel). Inset: higher magnification (40 ×) of the boxed areas

To determine whether adolescent exposure to JWH-018 was able to induce enduring changes in glial cells (astrocytes and microglia), we measured the levels of specific markers of neuroinflammation (IBA-1 and GFAP) in prefrontal cortex (PFC), NAc (shell and core), and caudate-putamen (CPu) of adult mice that underwent IVSA during adolescence. Two-way ANOVA of IBA-1 showed a main effect of treatment (*F*_(1,24)_ = 16,70, *p* = 0.0004), brain area (*F*_(3,24)_ = 8,176, *p* = 0.0006), but not brain area × treatment interaction. To evaluate the effect of adolescent JWH-018 IVSA on IBA-1 in each brain area, data were also analyzed separately. Student’s *t*-test showed increased expression of IBA-1, a marker of activated microglia within the NAc core and shell (*t*_(6)_ = 2.47; *p* = 0.0482, Cohen’ *d* = 1.7451; + 9%; *t*_(6)_ = 3.02; *p* = 0.0235, Cohen’ *d* = 2.0545; + 11% respectively) and CPu (*t*_(6)_ = 3.58; *p* = 0.0116, Cohen’ *d* = 1.1586; + 15%) of mice subjected to adolescent JWH-018 IVSA with respect to control (Veh); no significant differences in IBA-1 were observed in the PFC (Fig. 4(a)). Moreover, as shown in Fig. 4(c), IBA1 staining revealed that most of microglial cells displayed a rounded ameboid-like aspect, characteristic of activated microglia, while resting microglia in the NAc of control mice (Veh) showed a ramified appearance. Considering GFAP expression, two-way ANOVA showed a main effect of brain area (*F*_(3,25)_ = 38,76, *p* < 0.0001), treatment (*F*_(1,25)_ = 12,68, *p* = 0.0015), but not brain area × treatment interaction. However, analyzing each brain region separately, Student’s *t*-tests detected a significant decrease of GFAP levels in the CPu of JWH-018 mice compared to Veh group (*t*_(6)_ = 2.92; *p* = 0.0268, Cohen’ *d* = 2.066; − 29%); a downward trend, but non-significant, of GFAP expression was also observed in all the other analyzed brain areas in JWH-018 mice indicating possible astrocytopathy (Fig. 4(b)). Taken together, our data revealed an association between adolescent IVSA of JWH-018 and long-term microglia activation as well as potential effects on astrocyte density.

#### Adolescent JWH-018 IVSA results in altered cytokine expression in the brain of adult mice

**Fig. 5:**
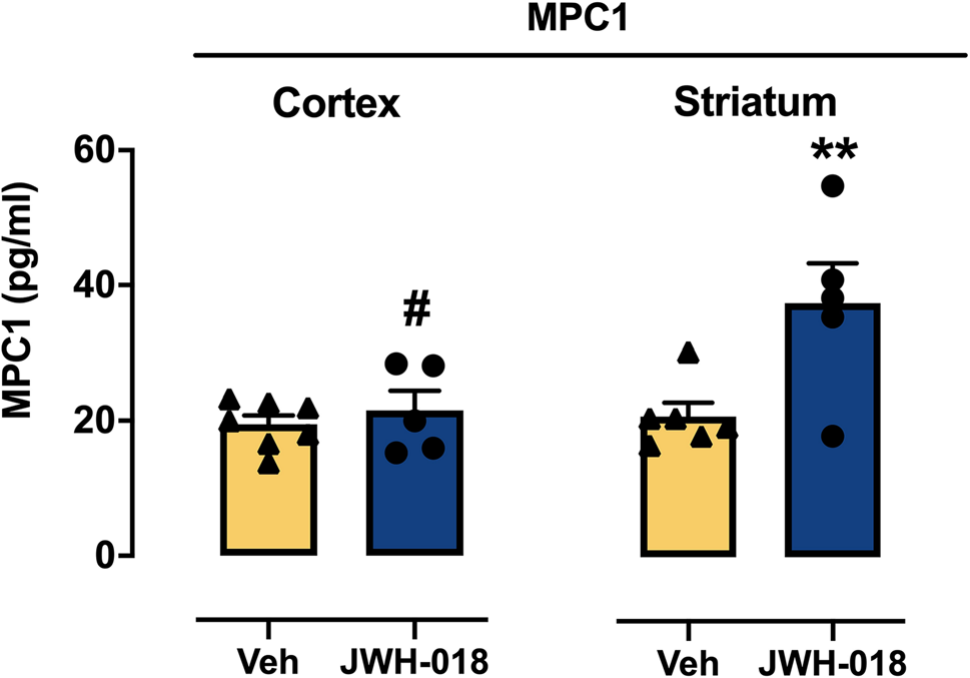
Levels of chemoattractant protein MPC1 in the cortex and striatum of adult mice after adolescent exposure to JWH-018. Adolescent JWH-108 IVSA significantly increased the levels of MPC1 in the dorsal striatum at adulthood. The increase of MPC1 is higher in the striatum than in the cortex of JWH-018 mice. Data are expressed as mean ± SEM of MPC1 levels, expressed as pg/ml. **p < 0.01 compared to Veh group; # *p* < 0.01 compared to striatum of JWH-018 group (two-way ANOVA; Tukey’s post hoc test); JWH-018: *n* = 5; Veh: *n* = 7

**Fig. 6:**
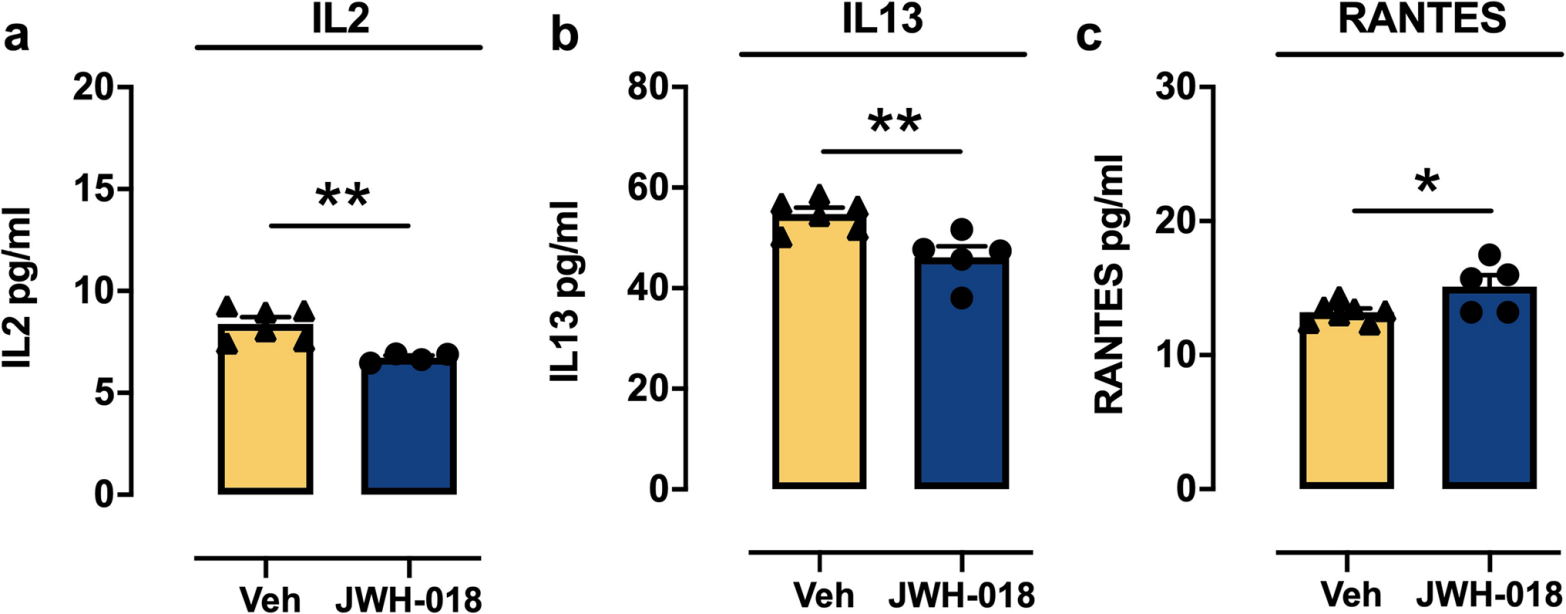
Multiplex analysis of cytokines in the cortex of adult mice after adolescent exposure to JWH-018. Lower levels of (**a**) IL2, (**b**) IL13, and (**c**) higher levels of RANTES were detected in the cortex of adult mice following adolescent IVSA of JWH-018. Data are expressed as mean ± SEM for each cytokine, expressed as pg/ml. *p < 0.05 and **p < 0.01 compared to Veh group (unpaired Student’s *t*-test); JWH-018: *N* = 4 (IL2), *N* = 5 (IL13, RANTES); Veh: *n* = 6 (IL2, IL13), *n* = 7 (RANTES)

We investigated the long-term effects of JWH-018 IVSA during adolescence on the concentration of cytokines/chemokines in the brains of adult mice using a fully quantitative ELISA-based chemiluminescent assay. Two-way ANOVA (treatment × brain area) for the chemoattractant protein MPC1 showed significant main effect of treatment (*F*_(1,19)_ = 8,878, *p* = 0.0077), of brain area (*F*_(1,19)_ = 7,813, *p* = 0.0115), and of treatment × brain area interaction (*F*_(1,19)_ = 5366, *p* = 0.0319). Tukey’s post hoc revealed a significant increase of MCP1 (*p* < 0.01; + 80%, Hedges’ *g* = 1.7416) in striatum of JWH-018 group compared to the control group (Veh). Moreover, the increase of this chemoattractant protein was significantly higher (*p* < 0.05, Cohen’ *d* = 1.5415; + 75%) in the striatum than in the cortex of JWH-018 IVSA mice (Fig. 5). On the contrary, two-way ANOVA for IL2, IL13, and RANTES showed a significant main effect of brain area [IL2 (*F*_(1,14)_ = 7109, *p* = 0.0184; IL13 (*F*_(1,17)_ = 19,712, *p* = 0.00035); RANTES (*F*_(1,21)_ = 38,764, *P* = 0.000004)], but no effect of treatment nor treatment × brain area interaction for any cytokine analyzed. Subsequently, data were analyzed separately using a Student’s *t*-test. As shown in Fig. 6(a)-(c)), adolescent JWH-018 IVSA induced a significant decrease in the cortex of IL2 (*t*_(8)_ = 3.99; *p* = 0.004, Hedges' *g* = 2.5721; − 20%) and IL13 (*t*_(9)_ = 3.44; *p* = 0.007, Hedges’ *g* = 2.0856; − 16%) levels, while RANTES level was significant increased (*t*_(10)_ = 2.50; *p* = 0.031, Hedges’ *g* = 1.4680; + 15%). Moreover, in the cortex, we observed an upward trend of IL1α, IL3, IL-4, IL5, IL17α, KC, and MCP-1 levels, a downward trend of IL2 levels, with no variation of IL1β, IL6, IL9, IL10, IL12 (p40), IL12 (p70), IFNγ, and Eotaxin expression, and undetectable TNFα and G-CSF at 300 µg proteins/well (see Table 1 for details). In the striatum, we observed a downward trend of IL2, IL3, IL13, IL17α, MIP1α, and MIP1β expression, no variation in IL1α, IL1β, IL4, IL5, IL6, IL9, IL10, IL12 (p40), IL12 (p70), KC, RANTES, and G-CSF, and undetectable TNFα and GM-CSF at the same concentration reported above (see Table 1 for details).

**Table 1:** Cytokine, chemokine, and colony-stimulating factor concentrations in the cortex and striatum of adult mice after adolescent JWH-018 or Vehicle IVSA. Data, expressed as a pg/ml, are mean ± SEM calculated from two independent experiments performed in duplicate. *ND*, not detectable: cytokines, chemokines, or colony-stimulating factors under the limit of detection. *p < 0.05 and **p < 0.01 compared to Veh group (unpaired Student’s *t*-test). For MCP-1 (two-way ANOVA; Tukey’s post hoc test). **p < 0.01 compared to Veh group; # *p* < 0.05 compared to striatum; *n* = 5–6 per group

	Cortex			Striatum		
	Veh	JWH-018	% change	Veh	JWH-018	% change
Cytokine						
IL1α	1.22 ± 0.06	1.36 ± 0.13	+ 11	2.23 ± 0.29	2.24 ± 0.43	-
IL1β	10.97 ± 0.60	10.94 ± 1.07	-	14.38 ± 0.80	13.99 ± 2.95	-
IL2	**8.39 ± 0.33**	**6.72 ± 0.11**	- 20**	17.02 ± 3.14	11.81 ± 4.86	- 31
IL3	5.39 ± 0.37	6.22 ± 0.67	+ 15	15.21 ± 0.38	12.87 ± 1.94	- 16
IL4	4.00 ± 0.27	4.67 ± 0.46	+ 16	9.35 ± 0.75	8.36 ± 1.46	-
IL5	1.51 ± 0.10	1.73 ± 0.17	+ 14	3.61 ± 0.23	3.66 ± 0.58	-
IL6	2.55 ± 0.11	2.62 ± 0.10	-	5.26 ± 0.37	4.98 ± 0.74	-
IL9	20.53 ± 0.47	19.53 ± 0.74	-	30.78 ± 1.59	29.46 ± 3.93	-
IL10	11.06 ± 0.48	11.73 ± 0.71	-	17.67 ± 1.49	16.85 ± 3.03	-
IL12(p40)	38.67 ± 1.83	37.79 ± 1.16	-	43.17 ± 2.37	41.19 ± 2.59	-
IL12(p70)	73.08 ± 5.60	85.63 ± 5.14	+ 17	163.70 ± 3.90	176.00 ± 18.05	-
IL13	**54.71 ± 1.31**	**46.13 ± 2.24**	- 16**	83.05 ± 3.99	69.11 ± 11.16	- 17
IL17	27.48 ± 1.86	30.33 ± 2.43	+ 10	56.63 ± 1.06	46.97 ± 7.15	- 17
IFNγ	26.29 ± 0.46	25.46 ± 0.99	-	42.39 ± 2.37	37.61 ± 6.04	- 12
TNFα	ND	ND		ND	ND	
Chemokine						
Eotaxin	31.94 ± 1.20	31.31 ± 2.10	-	70.52 ± 3.79	54.42 ± 7.70	- 23
KC	20.18 ± 1.12	22.67 ± 2.21	+ 12	34.79 ± 2.15	32.47 ± 5.08	
MCP1	19.46 ± 1.32	21.55 ± 2.87	+ 11	**20.97 ± 2.01**	**37.73 ± 5.97**	+ 80**^#^
MP1a	4.85 ± 0.70	4.53 ± 0.26	-	6.24 ± 0.89	5.19 ± 0.70	- 17
MP1ß	18.99 ± 0.62	20.53 ± 0.79	-	26.91 ± 0.58	23.82 ± 2.78	- 12
RANTES	**13.21 ± 0.27**	**15.12 ± 0.84**	+ 15*	32.94 ± 3.02	32.56 ± 5,45	-
Colony-stimulating factor						
GM-CSF	5.46 ± 0.40	5.89 ± 0.67	-	ND	ND	
G-CSF	ND	ND		7.16 ± 0.45	7.10 ± 0.51	-

Numbers in bold indicate significative difference between the two groups

#### Effect of adolescent JWH-018 IVSA on COX-2, EEAT2, and CB1Rs in specific brain areas of adult mice

**Fig. 7:**
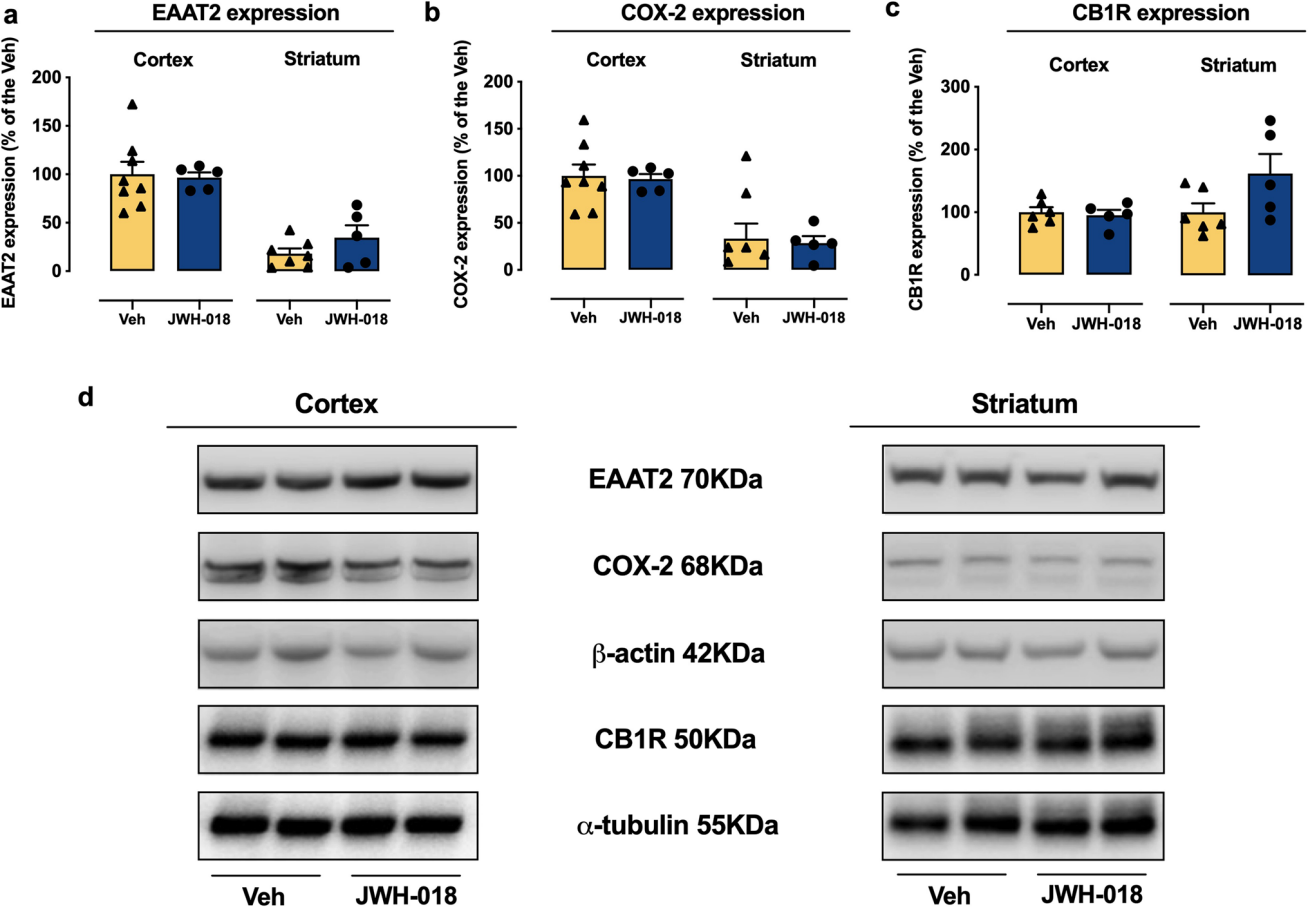
EAAT2, COX-2, and CB1R expression in the cortex and striatum of adult mice. The protein levels of (**a**) EAAT2, (**b**) COX-2, and (**c**) CB1R were quantified and normalized to loading control beta-actin for COX-2, EEAT2, and alpha-tubulin for CB1R, respectively, in cortex and striatum samples from adult mice after adolescent IVSA of JWH-018 or Veh (**d**) representative illustration of Western blotting analysis. Values represent mean ± SEM of EAAT2, COX-2, and CB1R levels expressed as percentage of Veh, normalized to β-Actin and α-tubulin levels for EAAT2, COX-2, and CB1R, respectively (unpaired Student’s *t*-test); EEAT2, JWH-018: *n* = 5 (cortex, CPu); Veh: *n* = 8 (cortex), *n* = 7 (CPu); COX-2, JWH-018: *n* = 5 (cortex, CPu); Veh: *n* = 8 (cortex), *n* = 7 (CPu); CB1R, JWH-018: *n* = 5 (cortex, CPu); Veh: *n* = 6 (cortex, CPu)

Microglia activation and altered function of astrocytes are often associated with markers of inflammation such as Cox-2 (Crews et al. 2011; Cutando et al. 2013; Zamberletti et al. 2015) and modified expression of the astrocyte glutamate transporter, EEAT2 (Kim et al. 2018; Wu et al. 2008). Therefore, we investigated the expression of Cox-2 and EEAT2 in cortex and striatum of adult mice that underwent to JVH-018 IVSA during adolescence. As shown in Fig. 7(a) and (b), no significant differences in EEAT2 and Cox-2 levels between groups were observed in either the cortex or striatum. Moreover, considering the crucial role of CB1Rs in brain areas involved in addiction and motivated behaviors (López-Gallardo et al. 2012; Parsons and Hurd 2015; Zamberletti et al. 2015) and that IVSA of JWH-018 in adolescent mice is mediated by the activation of CB1Rs (Fig. 1(f)), we examined whether changes in CB1Rs expression were present in the adult cortex and striatum following adolescent JWH-018 IVSA. As shown in Fig. 7(c), no significant differences were observed in the cortex and striatum, although there is an upward trend of CB1R levels in the striatum of JWH-018 mice.

## Discussion

A major aim of the present study was to develop and characterize an animal model suitable for investigating the reinforcing properties of SCRAs during adolescence as well as their behavioral and neurochemical long-term consequences at adulthood. Overall, the present study demonstrates, for the first time, that adolescent voluntary consumption of the prototypical SCRA JWH-018 alters repetitive/compulsive-like behavior, remodels glial cells, and affects cytokine expression in the brains of adult male mice.

The first key finding revealed by our investigation is that adolescent male mice (i) acquired operant behavior (lever pressing) for JWH-018 with an inverted U-shaped dose–response curve; (ii) that this behavior was specifically directed at obtaining JWH-018 since it increased under a Progressive Ratio schedule of reinforcement, and was absent in vehicle mice; (iii) that JWH-018 IVSA was reduced by pretreatment of the CB1-antagonist/inverse agonist AM251. Specifically, results showed that CD1 adolescent mice acquired JWH-018 IVSA at the dose of 7.5 µg/kg/inf (Fixed Ratio 1–3), displaying higher sensitivity to this agent compared to that shown by C57BL/6 (C57) adult mice (De Luca et al. 2015), thereby confirming the reinforcing effects of SCRAs identified in Spice/K2 products (De Luca et al. 2016; Musa et al. 2020). Of note is that we established a self-administration protocol for JWH-018 using a less sensitive strain of mice, which implies greater reinforcing properties of this particular SCRA with respect to those of previous generations (Martellotta et al. 1998; Navarro et al. 2001), and with respect to THC (Tanda and Goldberg 2003; Justinova et al. 2005). We observed that as the FR for JWH-018 was increased, the difference between active and inactive lever presses became significant and that this behavior was absent in the vehicle group. Importantly, the operant behavior was specifically directed at obtaining JWH-018, since it dramatically increased under the PR schedule of reinforcement. In contrast with what we observed with increasing doses of JWH-018 under FR1, the dose of 7.5 µg/kg/inf under a varying schedule of responding (from FR1 to FR3) hampered, unexpectedly, the acquisition process. A possible explanation is that JWH-018 intake under FR1 facilitates the acquisition process. In fact, previous studies on SCRA reported that an acute administration of WIN 55, 212–2 performed 24 h before the first session, acted as promoting factor for a faster SA acquisition of the same drug (Mendizabal et al. 2006). JWH-018 IVSA was reduced by the CB1R antagonist/inverse agonist AM251, but not by the CB2R antagonist/inverse agonist AM630. Moreover, in our experimental conditions, JWH-018 mice were not able to extinguish the operant behavior most likely due to spontaneous withdrawal signs (see Pintori et al. (2021) for description) that may drive to repetitive/persistent drug-seeking behavior and compulsive searching for the SCRA, as previously described by our group (De Luca et al. 2015). The resistance to extinction of instrumental responding after acquisition of JWH-018 IVSA (at least up to 6 consecutive sessions) could be related to the acquisition of a habit modality, consistent with a role of cannabinoids in habit learning formation (Hilàrio et al. 2007; Goodman and Packard 2015).

Moreover, in the present study, we reported that adolescent exposure to JWH-018 by IVSA induces repetitive/compulsive-like behaviors at adulthood, as revealed by the increase of repetitive/compulsive-like phenotypes both in the nestlet shredding and marble burying tests. Consistent with the present observations, adolescent mice treated with the SCRA 5F-MDMB-PICA for 14 consecutive days displayed repetitive/compulsive-like states at adulthood, as indexed by heightened perseverative responses in the marble burying test (Musa et al. 2020). Accordingly, Murphy et al. (2017) demonstrated that chronic administration of THC during adolescence increased nestlet shredding during adulthood; however, adolescence animals showed a trend to statistical significance in burying marbles only immediately after THC treatment. This discrepancy could be due to the different test lengths (i.e., longer observation time in our study), and to the higher potency of JWH-018 compared to THC. It is noteworthy that marble-burying test is also often employed to assess anxiety-like behavior, as marble-burying behavior by rodents may respond to compounds with anxiolytic activity (de Brouwer et al. 2019; Thomas et al. 2009). Although in this study we did not evaluate the predictive validity of the model by testing anti-compulsive and/or anxiolytic drugs, our previous investigations (Pintori et al. 2021) and the phenotypes observed in the nestlet shredding test indicate that the heightened marble burying activity of JWH-018-treated mice likely reflects repetitive/compulsive-like rather than anxiety-like states. Previous studies have reported that chronic use of Cannabis is associated with increased risk-taking as well as impulsive behavior and poor inhibitory control (Lane et al. 2005; Wrege et al. 2014), both in adolescent and young adult individuals (Claus et al. 2018; O’Donnell et al. 2021). To evaluate the risk propensity of JWH-018 exposed mice, we tested them on the wire-beam bridge, a reliable test able to assess risk-taking behavior in both rats and mice, and particularly susceptible to pharmacological manipulations of the dopamine (Bortolato et al. 2009; Frau et al. 2017; Frau et al. 2019; Festucci et al. 2021) and cannabinoid systems (Frau et al. 2019). However, using this test, we found that the adolescent exposure to JWH-018 did not elicit significant engagement in risky behaviors at adulthood. Although risk-taking propensity (i.e., an increase in substance use and risky sexual behaviors) has been observed in human adolescents exposed to SCRAs (Clayton et al. 2017), other clinical studies have shown that adolescent Cannabis users can recover cognitive deficits by reducing Cannabis use after 2 years from the beginning of the experiment (Becker et al. 2018). Therefore, while we used only one test to assess risk propensity, our findings suggest that risky behaviors may not be a persistent neurobehavioral outcome of the adolescent exposure to JWH-018.

A critical finding of our study was that adolescent voluntary consumption of JWH-018 alters microglia, astrocytes, and cytokine/chemokine expression in the brain of adult mice. As reported in the introduction, although growing evidence strongly supports a role for inflammatory changes in the pathophysiology of drug addiction disorders, the exact involvement of different pro-inflammatory or anti-inflammatory cytokine/chemokine produced either by microglia or astrocytes is unclear. Based on this knowledge, we decided to use a new tool such as the multiplex assay that maximizes cytokine/chemokine simultaneous detection in a single sample. Specifically, we demonstrated that JWH-018 IVSA induces at adulthood (i) an increased number of IBA1 positive cells and decreased GFAP expression in specific brain regions, (ii) increased levels of the chemoattractant protein MPC1 in the striatum, RANTES in cortex, and a decrease of IL2 and IL13 in cortex. In agreement with our data, Pintori et al. (2021) demonstrated that important features of the sequelae of JWH-018 exposure at adulthood are both astrogliosis and microgliosis. Despite the consistency of data showing a close correlation between neuroinflammation (i.e., gliosis, microglia activation) and drug addiction (Kim et al. 2018; Lacagnina et al. 2017; Pekny et al. 2016), the long-term effects of SCRA IVSA on glia have not been reported so far. Only two studies have shown that SCRAs induce apoptosis in forebrain neuronal cultures (Tomiyama and Funada 2014) and increase number of neural cells with distorted and pyknotic nuclei in the NAc and hippocampus, suggesting neurotoxic effects (Cha et al. 2015). Here, we demonstrated for the first time that JWH-018 IVSA during adolescence increased IBA-1 positive cells in the NAc shell and core and in the CPu, together with a decrease of GFAP levels in the CPu at adulthood. Images of IBA-1 staining revealed that cells show an ameboid-like appearance, indicative of an activated state of microglia. Conversely, in adult control mice microglia displayed small soma and ramifications with non-overlapping processes, phenotypes typical of “resting” or “surveillant” microglia (Kim et al. 2018). Accordingly, recent studies reported that in female rats, passive administration of THC during adolescence exerts at adulthood a persistent neuroinflammatory profile in the PFC associated with cognitive impairment and depressive-like behaviors (Zamberletti et al. 2015; Gabaglio et al. 2021); Lopez-Rodriguez et al. (2014) reported an increase of reactive microglial cells in the hippocampus of males, whereas an opposite trend was found in females following THC treatment. In addition, preclinical studies confirm that methamphetamine increases GFAP levels in PFC and CPu (Castelli et al. 2014) along with increased striatal mRNA expression levels of IL6 family pro-inflammatory cytokines (Robson et al. 2013). Taken together, our data confirm the association between adolescent cannabinoid exposure and altered levels of microglia cells in adult brains. The discrepancies observed among the studies could depend on the type of cannabinoids administered (natural or SCRA), the type of administration (passive or active exposure), sex of the animals, brain areas examined, and the methodologies used to investigate the number of IBA-1 positive cells (western or immunohistochemical assay). Interestingly, immune response dysfunctions and microglia dysregulation have been related to many psychiatric disorders including obsessive–compulsive spectrum disorders (Rocha et al. 2008; Kronfol and Remick 2000; Crews et al. 2011). Consistent with this idea, our study showed both an increase in repetitive/compulsive behaviors and an increase in IBA-1 levels in CPu, which is included in “OCD brain circuits” (Greer and Capecchi 2002).

Alterations in the endocannabinoid system due to adolescent THC exposure could lead to modifications in immune responses, such as cytokine expression (Bisogno and Di Marzo 2010; Stella 2010). In the present study, we detected all the cytokines/chemokines that we analyzed except for TNFα, GM-CSF (striatum), and G-CSF (cortex). However, we did not find any variations in IL1β, IFNγ, and IL10 between JWH-018 IVSA and vehicle mice. In contrast, an increase in TNFα and a decrease of IL10 in the hippocampus were observed by Zamberletti et al. (2016) in adult male and female rats treated with THC during adolescence, with the similar effects reported in the PFC of female, but not male, rats (Zamberletti et al. 2015; 2016). In the cortex of adult mice exposed to JWH-018 as adolescents, we found an increasing trend in IL4 levels and an increase in RANTES, a chemotactic cytokine that directs leukocytes to sites of inflammation and infection, along with a decrease in IL2 and IL13 expression compared to vehicle mice. Accordingly, studies performed in Cannabis and 3,4-methylenedioxy-methamphetamine (MDMA) human users showed decreased IL2 expression in blood samples (Pacifici et al. 2007). The same group showed that long-term exposure to Cannabis decreased IL2 levels, which in turn was associated with a decrease of an inflammatory response (Pacifici et al. 2002, 2003). We also observed increased MCP1 levels in the striatum of JWH-018 mice, in line with studies reporting that other drugs of abuse, such as ethanol, increased MCP1 (Zou and Crews, 2010; Crews et al. 2011). Taken together, data suggest that JWH-018 IVSA during adolescence could lead to an altered immunological response that persists into adulthood. Indeed, Kevin et al. (2017) reported no detection in plasma of GM-CSF and TFNα in adult rats after adolescent treatment with the SCRA AB-PINACA and AB-FUBINACA. However, contrary to our results, they found decreased IL1α and IL12 after AB-FUBINACA but not AB-PINACA.

Our study showed a decrease of GFAP expression in the CPu and a downward trend also in the NAc shell and core, providing direct evidence that adolescent JWH-018 IVSA may result in a reduction of GFAP that persisted later in life. The JWH-018-induced decrease of GFAP expression is likely brain region-specific, since no alterations were detected in the PFC of JWH-018 compared to vehicle mice. Accordingly, it has been reported that THC inhibits astroglia growth in vitro (Tahir et al. 1992) and affects their development in vivo (Suàrez et al. 2000). However, following adolescent treatment with THC, conflicting results have been reported regarding the levels of GFAP, with studies showing no changes, increased or decreased levels (Lopez-Rodriguez et al. 2014; Zamberletti et al. 2016). Of note, responsiveness of astrocytes to insults (e.g., injuries, inflammation, neuropsychiatric disease, drugs of abuse) ranges from astrocytopathy to astrogliosis and is characterized by several phenotypes including increased or decreased levels of GFAP and morphological features (i.e., hypertrophy/atrophy) (Kim et al. 2018). Consistently, GFAP expression and the number of astrocytes were reduced in the prelimbic cortex of ethanol-preferring rats (Miguel-Hidalgo 2005) as well as following prolonged exposure to alcohol (Franke 1995; Rintala et al. 2001).

As altered function of astrocytes and/or activated microglia in the CNS are often associated not only with modified levels of cytokines/chemokines but also with changes in the levels of iNOS and COX-2 (Crews et al. 2011; Cutando et al. 2013; Zamberletti et al. 2015) and/or decreased levels of GFAP, Aquaporin 4 and EAAT2 (Pekny et al. 2016), we measured the expression of EAAT2, an astrocyte transporter that removes synaptic glutamate in physiological conditions, in the striatum and the cortex. Down-regulation or up-regulation of EAAT2 has been reported after heroin, nicotine, cocaine, and alcohol exposure (Rao et al. 2015; Smith et al. 2015; Linker et al. 2018; Namba et al. 2020). We observed no difference in EAAT2 expression after adolescent exposure to JWH-018 in line with Higuera-Matas et al. (2012) showing no difference in cDNA EAAT2 levels in the hippocampus of adult rats treated during adolescence with the SCRA CP55,940. We also analyzed the expression of COX-2, the inducible isoform of COX, involved in inflammatory processes and prostaglandin production (Fishbein-Kaminietsky et al. 2014). Adolescent treatment with THC increased COX-2 levels in the PFC of male and female adult rats, but not in the hippocampus of male rats (Zamberletti et al. 2015; 2016). However, we found no difference in COX-2 expression in both cortex and striatum of adult mice following JWH-018 IVSA during adolescence. Despite the small size of our experimental groups, our neurochemical results might be representative of population investigated as also pointed out by the effect size for these results found to exceed Cohen’s or Hedge’s convention for a large effect (*d* = 0.80). Thus, the present results show not only statistical but also practical significance and application.

Finally, we evaluated the expression of CB1R in specific brain areas involved in addiction and motivated behavior (Parsons and Hurd 2015). We found no changes in the CB1Rs expression in the adult cortex or striatum of mice exposed to JWH-018 during adolescence. Different studies have reported that THC administration induced no change (Ellgren et al. 2007) or a permanent down-regulation of CB1R binding density within the amygdala and hippocampus of male (Rubino et al. 2008) and the PFC of female rats (Gabaglio et al. 2021). In addition, López-Gallardo et al. (2012) reported that chronic treatment with the SCRA CP55,940 during adolescence decreased CB1Rs expression in the hippocampus of adult male rats. However, in agreement with our data, chronic THC treatment during adolescence produced no difference in CB1R expression in the PFC of female rats (Zamberletti et al. 2015). Overall, the differences reported by studies investigating CB1R expression and function following cannabinoid administration could be ascribed to the different protocols and methods used.

These intriguing findings warrant further investigations to overcome sex differences in the recurrent use of JWH-018. Nevertheless, our model represents a useful tool to characterize the brain pathways involved in pharmacological and toxicological sequelae of SCRAs use that is a public health concern.

## Supplementary Information

Below is the link to the electronic supplementary material.

Supplementary file1 (DOCX 20 KB)

Supplementary file2 (PDF 75 KB)

Supplementary file3 (PDF 3000 KB)
