# Correction to: Adolescent self‑administration of the synthetic cannabinoid receptor agonist JWH‑018 induces neurobiological and behavioral alterations in adult male mice

**DOI:** 10.1007/s00213-023-06353-3

**Published:** 2023-03-17

**Authors:** Giulia Margiani, Maria Paola Castelli, Nicholas Pintori, Roberto Frau, Maria Grazia Ennas, Antonio C. Pagano Zottola, Valeria Orrù, Valentina Serra, Edoardo Fiorillo, Paola Fadda, Giovanni Marsicano, Maria Antonietta De Luca

**Affiliations:** 1 Department of Biomedical Sciences, University of Cagliari, Cagliari, Italy; 2 “Guy Everett” Laboratory, Department of Biomedical Sciences, University of Cagliari, Cagliari, Italy; 3 Institute for Genetic and Biomedical Research, National Research Council (CNR), Lanusei, Italy; 4 INSERM, U1215 NeuroCentre Magendie, Bordeaux, France; 5 University of Bordeaux, Bordeaux, France; 6 Institute of Neuroscience‑Cagliari, National Research Council (CNR), Cagliari, Italy; 7 Institut de Biochimie et Génétique Cellulaires, UMR 5095, Bordeaux, France

**Correction to: Psychopharmacology**

**https://doi.org/10.1007/s00213-022-06191-9**

After publication of this paper, the authors inadvertently excluded Dr. Pagano Zottola from the list of authors and that the journal Psychopharmacology has no responsibility for the omission.

All authors agree to correct the list of authors as follows:

Giulia Margiani#,1, Maria Paola Castelli #,1, Nicholas Pintori1, Roberto Frau1,2, Maria Grazia Ennas1, **Antonio C. Pagano Zottola4,5,7**, Valeria Orrù3, Valentina Serra3, Edoardo Fiorillo3, Paola Fadda1,6, Giovanni Marsicano4,5,Maria Antonietta De Luca1

4 INSERM, U1215 NeuroCentre Magendie, Bordeaux, France

5 University of Bordeaux, Bordeaux, France

7 Institut de Biochimie et Génétique Cellulaires, UMR 5095, Bordeaux France

The original article has been corrected.

