# Supplementary material for: Adolescent self-administration of the synthetic cannabinoid receptor agonist JWH-018 induces neurobiological and behavioral alterations in adult male mice
Source: Psychopharmacology (Berl). 2022 Aug 9;239(10):3083–102. doi: 10.1007/s00213-022-06191-9 (PMC9481487; doi:10.1007/s00213-022-06191-9)
Supplement: Supplementary file 1 — Supplementary file1 (DOCX 20 KB) [file 213_2022_6191_MOESM1_ESM.docx]

**PSYCHOPHARMACOLOGY**

**Adolescent self-administration of the syntHetic CANNABINOID RECEPTOR AGONIST JWH-018 INDUCES neurobiological and behavioral alterations in adult MALE mice**

**Giulia Margiani*^1^, Maria Paola Castelli*^1^, Nicholas Pintori^1^, Roberto Frau^1,3^, Maria Grazia Ennas^1^, Valeria Orrù^2^, Valentina Serra^2^, Edoardo Fiorillo^2^, Paola Fadda^1^, Giovanni Marsicano^4,5^, and Maria Antonietta De Luca^1^**

Correspondence to:

Maria Antonietta De Luca, PhD

Department of Biomedical Sciences

University of Cagliari (Italy)

Phone: +390706758633

Email: [deluca@unica.it](mailto:deluca@unica.it)

**SUPPLEMENTARY INFORMATION**

**MATERIALS AND METHODS**

***Surgery***

On PND 25-26, mice were anesthetized with isoflurane gas, and maintained under anesthesia using a breathing tube under a scavenging system. Lubricant eye drops (Tears Naturale, Alcon, USA) was applied to both eyes to prevent them from drying out during the procedure. A chronic intravenous catheter for mice was prepared and implanted in the right jugular, as previously described (David et al., 2001; De Luca et al., 2015). After surgery, mice were housed individually under standard conditions (see *2.1*); in order to ensure full recovery, mice underwent post-operative antibiotic treatment with gentamycin sulphate (30 mg/kg, subcutaneously [s.c.]), for 3 days to aid recovery before starting the IVSA.

***IVSA experiments***

Daily IVSA sessions of 2h were carried out from 9 AM to 4 PM, in chambers housed in sound-proof boxes (model LE 1002 Panlab Harvard Apparatus, Spain). SA boxes were equipped with two levers, and the responses on one lever (active lever) delivers one reinforcer, whereas responses on the second lever had no programmed consequences (inactive lever). A white light was placed over the active lever as discriminative stimuli. Prior to each daily session, the jugular catheter was flushed with 0.1 ml of sterile saline and the mice were placed in the SA boxes. At the end of each SA session, the catheters were flushed with 0.1 ml of heparinized saline and the mice were returned to their home cages where a daily ratio of 4g of standard food was made available, which maintained body weights at stable levels throughout these studies. The responses performed by each mouse on both levers for the entire 2h daily session and the corresponding number of reinforcers received was recorded (Panlab software, Panlab Harvard Apparatus, Spain). In all the IVSA experiments performed, the patency of intravenous catheters was evaluated at the end of the experiments by an infusion of 0.05 ml of thiopental sodium (5 mg/mL) (Braun Medical) through the catheter. If the signs of anesthesia were not apparent within 3 seconds of the infusion, the mouse was removed from the experiment.

***Brain tissue preparation and GFAP and IBA-1 immunofluorescent staining***

Mice were deeply anaesthetized with Equithesin (0.97 g pentobarbital, 2.1 g magnesium sulphate, 4.25 g chloral hydrate, 42.8 mL propylene glycol, 11.5 mL ethanol 90%, 5 mL kg 1, intraperitoneal) and then transcardially perfused with 4% paraformaldehyde and 0.1% glutaraldehyde in 0.1 M phosphate-buffered saline (PBS, pH 7.4). Brains were rapidly removed and post-fixed in the same fixative for 6 h. After repeated washing in 0.1 M PBS, brains were cryoprotected in 30% sucrose in PBS for 48 h. Immunostaining was performed on free-floating coronal sections (thickness: 40 µm) which were obtained using a cryostat at levels containing the selected brain areas (PFC, CPu, NAc shell and core) according to the Atlas of Mouse Brain, Paxinos and Franklin, 2001. To facilitate the identification of the selected brain areas, adjacent sections were also collected and stained with Neutral Red. After repeated washing in 0.1 M PBS, tissue sections were incubated for 1 hour at 20°C in working solution of mouse immunoglobulin-blocking reagent, prepared as specified by the manufacturer (Vector Laboratories, Burlingame, CA). Following washing, pre-blocking of tissue sections was performed using normal goat serum (NGS, 5%) and normal donkey serum (NDS, 5%), bovine serum albumin (BSA, 1%) and Triton X-100 (0.2%) in PBS for 1 h at room temperature. As concerns GFAP-immunofluorescence, tissue sections were incubated at 4°C for 24 h with a mouse monoclonal anti-GFAP antibody (1:5000; Millipore Temecula, CA, USA) in PBS containing 0.2% Triton X-100, 0.1% BSA, 1% NDS. Then, sections were washed in PBS containing 0.2% Triton X-100 and incubated them with Alexa Fluor 594-labeled donkey anti-mouse IgG (1:500; Molecular Probes, Eugene, OR, USA) for 1 h in the dark at room temperature (Castelli et al., 2014). As concerns IBA-1 immunofluorescence, tissue sections, after washing, were incubated at 4°C for 24 h with a rabbit polyclonal anti-IBA-1 antibody (1:2000; Wako Pure Chemical Industries, Chuo-Ku, Osaka, Japan) in PBS containing 0.2% Triton X-100, 0.1% BSA, 1% NGS. Then, sections were washed in PBS containing 0.2% Triton X-100 and incubated them with Alexa Fluor 488-labeled goat anti-rabbit IgG (1:500; Molecular Probes, Eugene, OR, USA) for 1 h in the dark at room temperature. Moreover, standard control experiments were performed by omitting either the primary or secondary antibody and yielded no cellular labelling (data not shown).

***Imaging and quantitative analysis of GFAP and IBA-1 immunofluorescent staining***

For each animal, analysis of GFAP and IBA-1 immunoreactivity (IR) was performed on one tissue section out of every 2 successive sections for a total of 3 sections containing the PFC, one tissue section out of every 3 successive sections for a total of 4 and 10 sections containing the NAc (shell and core) and the CPu, respectively. The total size of the examined area in which GFAP-IR and IBA-1-IR cells were counted was selected according to the extension of the region under analysis, in order to include the whole area (either PFC, CPu, NAc core and shell). The selected coronal levels of these sections corresponded to the levels of plates 12-15 for the PFC (AP: +2.2 to +1.94), 21–31 for the CPu (AP: +1.18 to +0.02), 12-24 for NAc shell and core (AP: +1.18 to +0.86) (Atlas of Mouse Brain, Paxinos and Franklin, 2001). GFAP-IR analysis was carried out as previously described (Castelli et al., 2014). Briefly, three images of non-overlapping regions of interest (ROIs) from one out of either every 3 slices of NAc and CPu region or 2 slices of the PFC region were analyzed, and positively stained fibers were detected using the Cell P Analysis software module. The number of IBA-1 positive cells was counted bilaterally in different sections containing PFC, NAc and CPu sections per animal. In these sections, non-overlapping randomly selected ROIs of 0.15 mm^2^ (N=6) were examined with a 20X objective by two trained observers blind to drug treatment. Limits of the ROI were defined based on structural details within the tissue sections to ensure the ROIs did not overlap. The distance among the 6 ROIs was superior to 40 μm to avoid overlapping. IBA-1 positive cells touching the inferior or the right sides of the ROI were excluded from counting. GFAP-IR was expressed as average values of percentage of area occupied by fibers while the number of IBA-1 cells was expressed as mean/mm^2^.

***Sample processing***

Briefly, frozen tissues were thawed and disrupted in Bioplex cell lysis buffer (BioRad, Hercules, CA, USA) containing factors 1 and 2 (protease and phosphatase inhibitors, respectively; BioRad) and the protease inhibitor phenyl-methylsulfonyl fluoride (500 mM; Sigma–Aldrich). Samples were homogenized on ice with a potter (Quiminigen, Portugal) and homogenate frozen for 30 min at -80 °C. After thawed and sonicated for 15 sec, samples were centrifuged at 4°C and 4500 × g (Thermo Scientific Sorvall Legend Micro 17R Refrigerated Centrifuge) for 8 min. The supernatant was removed, and the protein content of each sample was determined using the BioRad Protein Assay (BioRad), with bovine serum albumin as a standard, according to the manufacturer’s protocol. The protein concentrations were determined according to Bradford method using Bio-Rad DC protein assay kit (Bio-Rad). Lysates were stored at -80°C until the day of experiment.

For CB1R immunoblotting, samples were homogenized in PBS pH 7.4 1X (Gibco by life technologies, Carlsbad, US) supplemented with protease and phosphatase inhibitors purchased from Roche (Basel, Switzerland), using the Tissue Lyser (Quiagen, Hilden, Germany). The protein contents were determined with Roti-Nanoquant protein quantitation assay, following manufacturer’s instruction (Carl Roth, Karlsruhe, Germany).

***Cytokine measurements***

Assays were performed in 96-well plates; briefly, each assay plate layout consisted of eight standards in duplicate, two blank wells and up to 39 mouse brain homogenate samples (diluted 1:3 prior to assay), run in duplicate at 300 μg proteins/well. Samples from Veh and JWH-018-treated animals were analyzed in the same plate. All the wash steps were performed on a Bio-Plex Pro wash station at room temperature. A Bio-Plex MagPix Multiplex System by Luminex was used to read the plate and data were analyzed using BioPlex manager 4.1 software with 5-parametric logistic regression (5PL) curve fitting to determine the standard curve (pg/ml) from 8 standards in duplicate and extrapolate the sample concentrations from the standard curve. Only standards and samples with coefficients of variance under 5% were used. When readings fell under the detection limit (below the background value) they were excluded from analyses.
