# Supplementary figures and images for: Adolescent self-administration of the synthetic cannabinoid receptor agonist JWH-018 induces neurobiological and behavioral alterations in adult male mice
Source: Psychopharmacology (Berl). 2022 Aug 9;239(10):3083–102. doi: 10.1007/s00213-022-06191-9 (PMC9481487; doi:10.1007/s00213-022-06191-9)

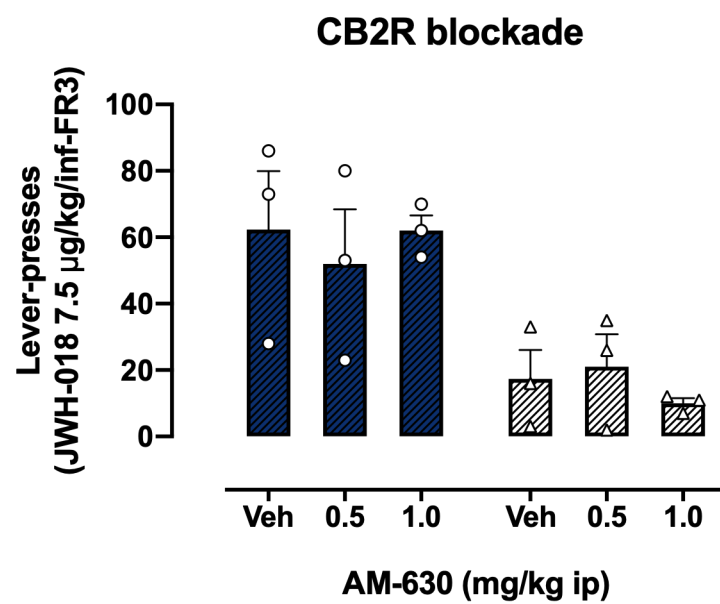

Supplement: Supplementary file 2 — Supplementary file2 (PDF 75 KB) [file 213_2022_6191_MOESM2_ESM.pdf]
