# Supplementary material for: Adolescent self-administration of the synthetic cannabinoid receptor agonist JWH-018 induces neurobiological and behavioral alterations in adult male mice
Source: Psychopharmacology (Berl). 2022 Aug 9;239(10):3083–102. doi: 10.1007/s00213-022-06191-9 (PMC9481487; doi:10.1007/s00213-022-06191-9)
Supplement: Supplementary file 3 — Supplementary file3 (PDF 3000 KB) [file 213_2022_6191_MOESM3_ESM.pdf]

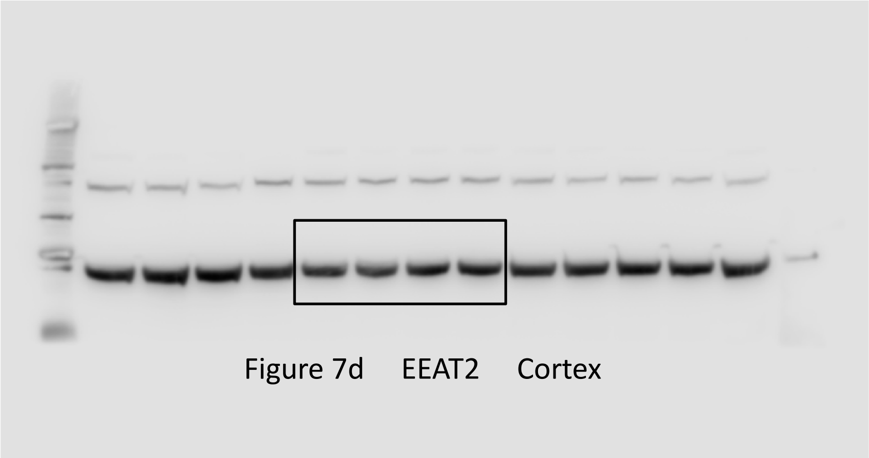

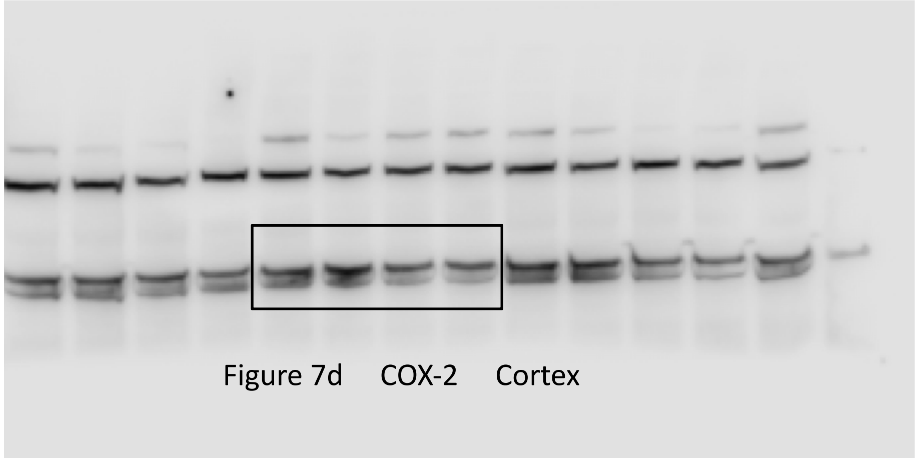

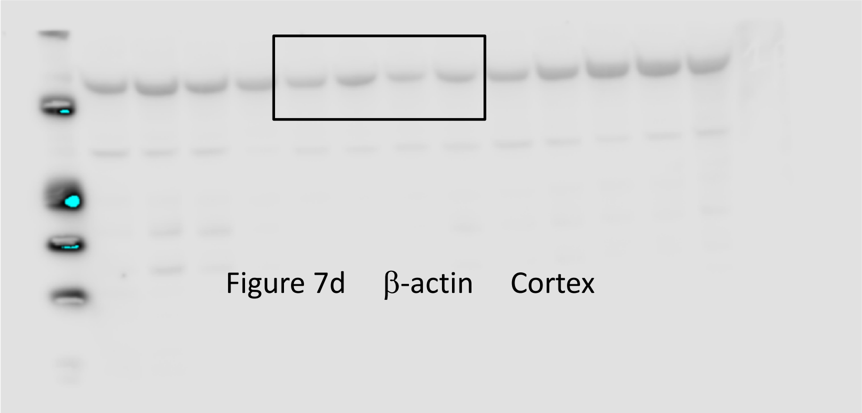

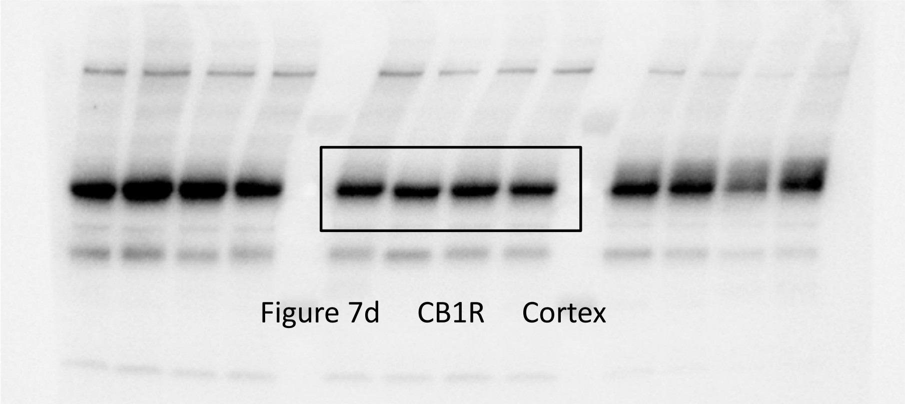

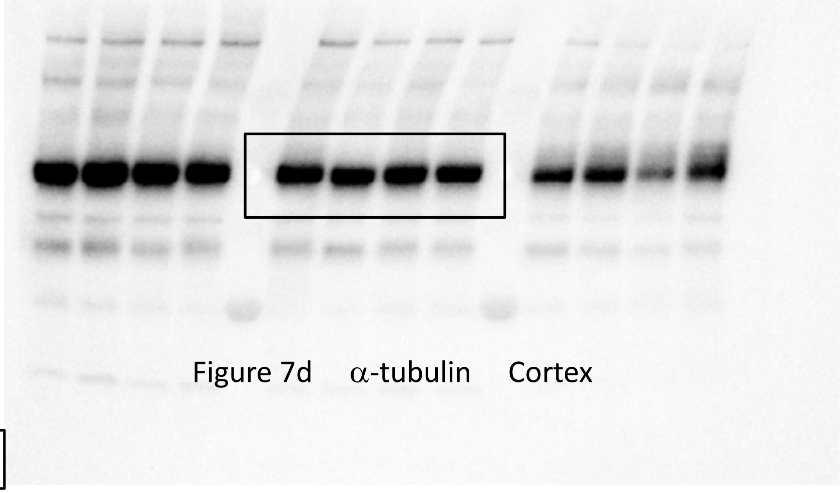

Supplemental Figure 1. Untruncated image of the blots shown in Fig. 7d.

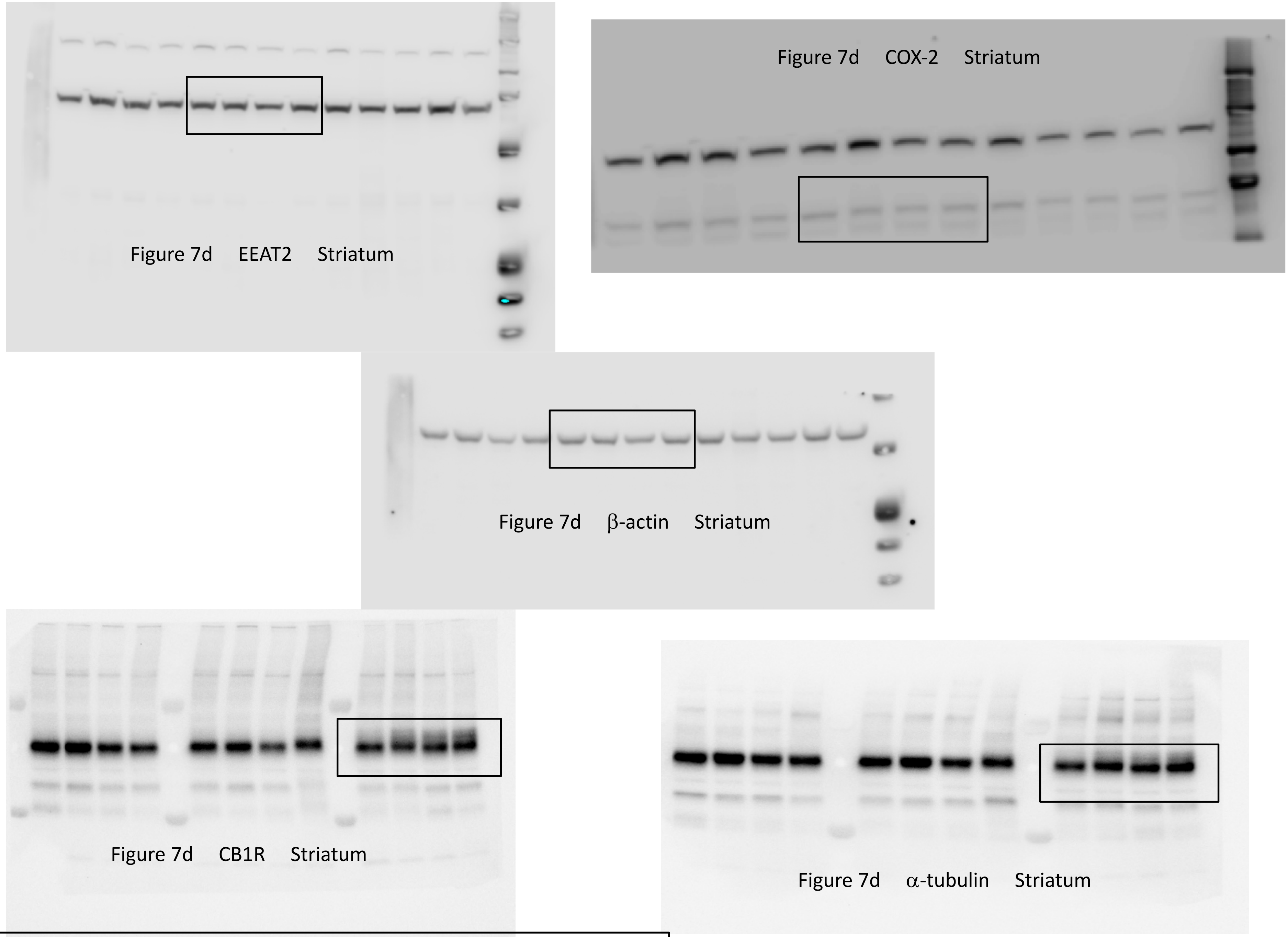

Supplemental Figure 1. Untruncated image of the blots shown in Fig. 7d.
